# GluN2A-NMDA receptor inhibition disinhibits the prefrontal cortex, reduces forced swim immobility, and impairs sensorimotor gating

**DOI:** 10.1038/s41401-025-01643-2

**Published:** 2025-09-10

**Authors:** Yuan-ping Dong, Yun Wu, Yi-lu Zhao, Yu-min Chen, Tong-ye Liu, Yi-he Zhang, Jie-ying Xie, Jin-feng Zhang, Han Zhang, He Chen, Yu Peng, Chun-lei Zhang, Andrew R. Rau, Kasper B. Hansen, Hai-bing Xu, Feng Yi

**Affiliations:** 1 Key Laboratory of Mental Health of the Ministry of Education, Guangdong-Hong Kong-Macao Greater Bay Area Center for Brain Science and Brain-Inspired Intelligence, Guangdong-Hong Kong Joint Laboratory for Psychiatric Disorders, Guangdong Province Key Laboratory of Psychiatric Disorders, Guangdong Basic Research Center of Excellence for Integrated Traditional and Western Medicine for Qingzhi Diseases, Department of Neurobiology, School of Basic Medical Sciences, Southern Medical University, Guangzhou 510515, China; 2 Department of Neurology, Guangzhou First People’s Hospital, School of Medicine, South China University of Technology, Guangzhou 510006, China; 3 Institut Pasteur, Université de Paris, Neural Circuits for Spatial Navigation and Memory, F-75015 Paris, France; 4 Center for Structural and Functional Neuroscience, Center for Biomolecular Structure and Dynamics, Division of Biological Sciences, University of Montana, Missoula, MT, USA

**Keywords:** NMDA receptor, psychiatry, prefrontal cortex, microcircuit, disinhibition

## Abstract

Recent investigations into the rapid antidepressant effects of ketamine, along with studies on schizophrenia-related susceptibility genes, have highlighted the GluN2A subunit as a critical regulator of both emotion and cognition. However, the specific impacts of acute pharmacological inhibition of GluN2A-containing NMDA receptors on brain microcircuits and the subsequent behavioral consequences remain poorly understood. In this study, we first examined the effects of MPX-004, a selective GluN2A NMDA receptor inhibitor, on behavior within the dorsomedial prefrontal cortex (dmPFC). Local administration of MPX-004 in the dmPFC led to a reduced immobility duration in the forced swim test, an acute antidepressant-like effect, impairments in sensorimotor gating, and a schizophrenia-like phenotype. In vivo multiple-channel recordings and c-Fos staining revealed that MPX-004 decreases the activity of parvalbumin-expressing interneurons (PV-INs) and increases the activity of pyramidal neurons (PYNs). In vivo patch-clamp recordings further confirmed that PV-IN inactivation leads to an elevated PYN firing rate in the PFC. In vitro whole-cell recordings demonstrated that PV-INs receive stronger excitatory synaptic input and respond more robustly to presynaptic stimulation than do somatostatin-expressing interneurons (SST-INs) and PYNs, rendering them susceptible to GluN2A inhibition. Finally, the specific knockdown of GluN2A in prefrontal PV-INs abolished the behavioral effects of MPX-004, underscoring a critical role of the GluN2A-mediated modulation of PV-INs in these phenotypes. Together, these findings reveal that PV-INs are particularly vulnerable to GluN2A inhibition, leading to disinhibition of prefrontal circuits and resulting in both antidepressant-like and schizophrenia-like behaviors.

## INTRODUCTION

Dysfunction of the medial prefrontal cortex (mPFC) is a central feature of many psychiatric disorders, including depression [1–3] and schizophrenia [4–8], which are observed in both humans and animal models [1–3, 5, 6]. The excitatory and inhibitory (E/I) balance within mPFC microcircuits is crucial for mPFC-related behaviors and psychiatric conditions [9, 10]. Among the various neuronal types in the mPFC, GABAergic interneurons play a significant role in maintaining E/I balance [11], which is essential for emotional regulation and cognitive processes [12, 13]. For example, inhibiting mPFC GABA interneurons has been linked to rapid antidepressant effects [14–16], and selective suppression of parvalbumin interneurons in the mPFC enhances resilience to chronic stress in mice [16]. Moreover, hypofunction of these interneurons is associated with schizophrenia pathology [17, 18]. Chemogenetic activation of parvalbumin interneurons alleviates cognitive deficits in schizophrenia mouse models [15]. Thus, understanding the mechanisms regulating E/I balance is essential for interpreting mPFC function and its role in psychiatric disorders.

Among the various factors that regulate mPFC microcircuits, NMDA receptors are crucial for fundamental cognitive functions, such as learning and memory [19–21]. The inhibition of NMDA receptors by MK801 disrupts microcircuits by disinhibiting prefrontal pyramidal neurons [22]. Dysregulation of NMDA receptor activity is linked to both depression and schizophrenia [5–7, 23–29]. Notably, ketamine, a well-known NMDA receptor antagonist frequently studied in schizophrenia research [30], has shown rapid and sustained antidepressant effects in clinical settings [31]. In addition to previously identified mechanisms involving GluN2B-containing NMDA receptors [32, 33] and non-NMDA receptor pathways [34, 35], emerging research indicates that GluN2A-containing NMDA receptors [36, 37] also play crucial roles in the antidepressant effects of ketamine. These findings underscore the potential therapeutic potential of selectively antagonizing GluN2A in treating depression [38].

In parallel, genetic investigations, including exome sequencing and genome-wide association studies, have pinpointed the *GRIN2A* gene, encoding the GluN2A subunit, as a significant risk factor for schizophrenia [39, 40]. Mouse models with *Grin2a* heterozygous null mutants (*Grin2a-KO* constitutively) exhibit schizophrenia-like phenotypes, including reduced activity in the prefrontal cortex and hippocampus, a hyperdopaminergic state in the stratum [41], and increased resting gamma oscillation power [42]. Mouse models with homozygous *Grin2a-KO* exhibit schizophrenia-related phenotypes, including hyperlocomotion, deficits in memory and impaired prepulse inhibition [43].

Although reduced prefrontal activity is commonly reported in patients with schizophrenia, early-stage unmedicated patients display elevated prefrontal connectivity [44]. Therefore, pharmacological manipulation is essential for gaining a deeper understanding of the acute effects of GluN2A inhibition on brain microcircuits and behaviors.

The lack of reliable inhibitors for GluN2A-containing NMDA receptors [45] has hindered pharmacological investigations. To address this, we utilized MPX-004, a potent and selective negative allosteric modulator of GluN2A [46, 47], to investigate the effects of GluN2A antagonism in the dorsomedial prefrontal cortex (dmPFC), a brain region implicated in both depression [1–3, 48] and schizophrenia [2, 8]. We first validated the strong inhibitory effects of MPX-004 on GluN2A-containing di- and triheteromeric NMDA receptors during synaptic-like activation. We then assessed the impact of MPX-004 on behavior and explored the underlying microcircuit mechanisms through both in vivo and in vitro investigations. Our findings illuminate the roles of GluN2A-containing NMDA receptors in the regulation of the E/I balance and demonstrate that targeted antagonism of GluN2A can alter local microcircuits in the dmPFC, potentially leading to antidepressant-like and schizophrenia-like behavioral phenotypes.

## MATERIALS AND METHODS

### DNA constructs

Rat cDNAs for GluN1–1a (GenBank accession no. U08261), GluN2A (D13211), and GluN2B (U11419) were provided by S. Heinemann (Salk Institute, La Jolla, CA, USA), S. Nakanishi (Osaka Bioscience Institute, Osaka, Japan) and P. Seeburg (University of Heidelberg, Heidelberg, Germany). The DNA constructs used for the expression of GluN1/2A_C1_/2B_C2_ receptors were designed using methods similar to those in previous studies [46, 49]. The DNA construct for GluN1 expression in HEK293T cells encodes enhanced green fluorescent protein (EGFP) inserted between the CMV promoter in pCI-neo and the ORF of GluN1 (i.e., EGFP and GluN1 were not expressed as a fusion protein). This DNA construct results in high expression of EGFP and low expression of GluN1 while maintaining a linear relationship between EGFP and GluN1 expression [50].

### Whole-cell patch-clamp recordings using HEK293T cells

HEK293T cells were seeded onto glass coverslips coated with poly-*D*-lysine (0.1 mg/ml) approximately 24 h prior to experimentation and were maintained in Dulbecco’s modified Eagle’s medium with GlutaMax-I and sodium pyruvate (GIBCO, Thermo Fisher Scientific, USA). The medium was supplemented with 10% dialyzed fetal bovine serum (GIBCO, Thermo Fisher Scientific, USA), 10 U/ml penicillin, and 10 mg/ml streptomycin (GIBCO, Thermo Fisher Scientific, USA). The cells were transfected using the calcium phosphate precipitation method with plasmid cDNAs encoding GluN1, EGFP, and GluN2 subunits at a 1:1 ratio. For experiments involving triheteromeric NMDA receptors, plasmid cDNAs for the GluN1–1a, GluN2A_C1_ and GluN2B_C2_ subunits were transfected at a 1:1:1 ratio. To avoid NMDA receptor-mediated cytotoxicity, the culture medium was supplemented with 200 μM *D*,*L*-2-amino-5-phosphonovalerate and 200 μM 7-chlorokynurenic acid. Experiments were conducted approximately 24 h after transfection.

Whole-cell voltage-clamp recordings were conducted using an Axopatch 200B system (Molecular Devices, Sunnyvale, CA) at room temperature (20 °C) with a holding potential of −60 mV, unless otherwise specified. Recording electrodes, with an open-tip resistance of 2–4 MΩ, were fabricated from thin-wall glass micropipettes (TW150F-4; World Precision Instruments, Sarasota, FL) using a horizontal puller (P-1000; Sutter Instruments, Novato, CA). The electrodes were filled with an internal solution containing 110 mM D-gluconic acid, 110 mM CsOH, 30 mM CsCl, 5 mM HEPES, 4 mM NaCl, 0.5 mM CaCl_2_, 2 mM MgCl_2_, 5 mM 1,2-*bis*(2-aminophenoxy)ethane-*N*,*N*,*N*’,*N*’-tetraacetic acid, 2 mM NaATP, and 0.3 mM NaGTP, adjusted to pH 7.35 with CsOH. The extracellular solution consisted of 150 mM NaCl, 10 mM HEPES, 3 mM KCl, 0.5 mM CaCl_2_, and 0.01 mM EDTA (pH 7.4 with NaOH). Rapid solution exchange was performed on lifted cells using a two-barrel theta-glass pipette controlled by a piezoelectric translator (MXPZT-300; Siskiyou Corporation, Grants Pass, OR). The 10%–90% open-tip solution exchange times were between 0.6 and 0.8 ms. Data analysis was restricted to cells with current responses of less than 1000 pA and a series resistance of less than 10 MΩ.

### Transgenic mice

PV-CRE (stock# 008069, Jackson Laboratories, USA) and SST-CRE (stock# 013044, Jackson Laboratories, USA) were maintained using method similar to those in previous studies. To visualize PV^+^ and SST^+^ cells in the prefrontal cortex, homozygous PV-CRE mouse lines were crossed with a homozygous Ai14 reporter line (stock# 007914; Jackson Laboratories) to drive the expression of the red fluorescent protein tdTomato, whereas homozygous SST-CRE mouse lines were crossed with a homozygous Rosa-YFP reporter line (stock# 007920; Jackson Laboratories, USA).

### Stereotaxic animal surgery for in vivo electrophysiological recordings

Four adult male mice were anesthetized with isoflurane (2%–4% in atm; HUAYON, China) and mounted in a stereotaxic frame. The mice were then implanted with custom-built electrode-cannula microdrives containing eight movable tetrodes. Each tetrode was assembled by twisting four 12.7-μm tungsten wires (California Fine Wire) that were then platinum-plated to achieve an impedance of approximately 200 kΩ. Recordings targeted the dorsomedial prefrontal cortex (dmPFC) in one hemisphere, positioned at the following stereotaxic coordinates relative to bregma: anterior-posterior (AP): +1.92 mm, medial-lateral (ML): +0.88 mm, and dorsal-ventral (DV): −1.81 mm. After surgery, the animals were allowed to recover for one week under standard housing conditions.

### In vivo multiple-channel recordings, spike sorting, and unit classification

Recordings were conducted while the animals were in their home cages (radius = 10 cm) lined with familiar soft towels. Following each recording session, all electrodes were lowered by 50–100 μm to record a new population of cells, with at least 48 h between recordings. In vivo electrophysiological recordings were performed using ultralightweight cables by the Intan recording system (RHD2000-Series Amplifier Evaluation System, Intan Technologies, USA) for 32-channel recordings at a sampling rate of 20 kHz. Eleven sessions involving 6 mice were carried out. Each session involved recording electric signals for 20 min after the infusion of 0.5 μL of vehicle, followed by an additional 20 min after the infusion of 0.5 μL of MPX-004. Vehicle or 10 μM MPX-004 was infused via a microsyringe pump at a rate of 0.05 μL/min. MountainSort4 was used for automatic spike sorting. Clusters were visually inspected and manually refined using Phy. Subsequently, CellExplorer was employed to classify cells based on the trough-to-peak latency, yielding narrow-spiking ( <0.4) and wide-spiking ( >0.4 ms) cell types [51–53].

### In vivo patch-clamp recordings

Surgeries were conducted under continuous isoflurane anesthesia, with 5% used for induction and 1%–3% used for maintenance. Mice received preoperative treatment with 0.1 mg/kg buprenorphine administered intraperitoneally and local lidocaine at 0.4 ml/kg in a 1% solution. The mice were secured in a stereotaxic apparatus, and a half-circle stainless steel headpost was affixed to the skull using dental cement. Following the procedure, the animals were allowed a two-week recovery period. Throughout the surgery, body temperature was monitored and maintained at 37 °C using a heating pad. Metacam was administered at 1 mg/kg intraperitoneally before the animals were returned to their home cages.

Circular craniotomies 0.5 mm in diameter were performed above the prefrontal cortex under isoflurane anesthesia approximately 1 h before recording. The stereotaxic coordinates relative to bregma were as follows: anteroposterior +2.7–3.1 mm and mediolateral ± 0.4–1.0 mm. A dental drill was used to create these precise openings.

For interneuron inactivation experiments, viral vector transduction involved injecting adeno-associated viral vectors into PV-Cre or SOM-Cre mice. The primary viral vector used was AAV5-hSyn-DIO-hM4D(Gi)-mCherry at a concentration of 7 × 10^12^/mL. The control viruses used included AAV1-CAG-FLEX-tdTomato-WPRE and AAV1-CAG-tdTomato-WPRE at concentrations of 1 × 10^13^/mL and 5 × 10^12^/mL, respectively. Viral vector injections were performed bilaterally into the prefrontal region of 6–8-week-old mice (AP: +2.7–3.1 mm and ML ± 0.4–0.6 mm from bregma) at an injection rate of 0.1 μL/min using glass pipettes and an oil-hydraulic micromanipulator. Each site received 0.3–0.4 μL of viral solution, with two injections made at depths of 300 and 500 μm from the dura. Headpost implantation occurred three weeks after the viral vector injections. Prior to recordings, clozapine-N-oxide (CNO) was administered intraperitoneally at 5 mg/kg to activate the hM4D receptor, with injections performed 30 min before recordings. All experimental recordings were completed within three hours of CNO administration.

Whole-cell patch-clamp recordings were conducted on head-restrained, awake mice were positioned on a treadmill apparatus. Recording pipettes were fabricated from borosilicate glass capillaries, resulting in pipettes with an approximate resistance of 5 MΩ. The internal pipette solution contained the following (in mM): 130 KCH_3_SO_3_, 7.0 KCl, 0.3 MgCl_2_, 0.1 EGTA, 10 HEPES, 1 sodium phosphocreatine, 3.0 Na_2_ATP, 0.3 NaGTP and 5 mg/ml biocytin (pH 7.2 adjusted with KOH, 289 mOsm).

The patch-clamp technique employed a standard blind-patch methodology. Pipettes were initially pressurized with high positive air pressure ( ~1000 millibars) and carefully positioned into the dorsal prefrontal cortical region through a small surgical opening ( ~500 micrometers) using a precision micromanipulator. The recording depths ranged from 150 to 850 micrometers beneath the pial surface. When the depth reached approximately 150 micrometers, the air pressure was reduced to 50–80 millibars. For quality control, cells with initial access resistances greater than 70 MΩ or greater than 100 MΩ during recordings were not included in the analyses. Recordings were made in current-clamp recording mode with no holding current, low-pass filtered at 10 kHz, and sampled at 50 kHz (Intan Technologies Clamp system). A silver/silver chloride reference electrode (0.3 mm in diameter) was positioned near the lambda suture point. The external perfusion solution contained (in mM) 150 NaCl, 2.5 KCl, 10 HEPES, 2 CaCl_2_, and 1 MgCl_2_ (pH 7.2, 289 mOsm) and was delivered through a circular plastic chamber.

### Animal maintenance for behavioral tests

In this study, male C57BL/6 J mice aged 4–8 weeks and weighing approximately 20 grams (Charles River, China) were used. The mice were housed in individually ventilated cages (IVCs) with two mice per cage under a 12-h light/dark cycle (lights on at 8:00 AM, lights off at 8:00 PM). Food and water were provided *ad libitum*. Prior to testing, the mice were handled daily for at least 3 days to acclimate them to the experimenter. On the day of testing, the mice were placed in the behavioral room for a minimum of 1 h to adapt to the new environment. After each behavioral test, the equipment was cleaned with 30% alcohol to eliminate residual odors.

### Stereotaxic animal surgeries guide cannula implantation

Eight-week-old male mice were deeply anesthetized with isoflurane and maintained under anesthesia throughout the surgery. The procedures were conducted using stereotaxic equipment (HUAYON, China). Each mouse was implanted with a unilateral guide cannula (RWD Life Science, China) for drug delivery into the prefrontal cortex at the following coordinates relative to bregma: AP: +1.70 mm, ML: +0.4 mm, and DV: −2.1 mm. The guide cannula had an outer diameter of 0.48 mm. After the surgery, the mice were returned to their home cages and allowed to recover for 7 days.

### Drug treatment for behavioral tests

MPX-004 (M-280, Alomone Labs, Israel) was prepared by dissolving it in DMSO to a stock concentration of 50 mM and subsequently diluting it to 10 μM with artificial cerebrospinal fluid (ACSF) containing 10% PEG400 to increase its solubility in the infusion solution. On the test day, mice with implanted guide cannulas received an injection of either 0.5 μL of vehicle control or 10 μM MPX-004 via a microsyringe pump at a rate of 0.1 μL/min. No perioperative analgesics were used during this procedure. After the injection, the injection needle was left in place for 5–10 min to prevent backflow before being slowly removed. The open field test (OFT), prepulse inhibition (PPI) test, and forced swim test (FST) were conducted on separate days during the daytime, with each behavior test performed 30 min after injection.

### Open field test

Locomotion was assessed using the open field test (Omnitech Electronics Superflex, USA) conducted over a 30-min period. The test setup included eight measurement boxes, each measuring 40 × 40 × 30 cm, which were equipped with sensors to record movement distances. Mice were individually placed in the center of a box at the start of the experiment. Movement was tracked using an automated system and recorded with Fusion software, which captures data for each cage event. The analysis included the cumulative distance traveled every 5 min and the total distance traveled over the entire 30-min period.

Anxiety levels were assessed by measuring the amount of time the mice spent in the center of the open field during the first ten minutes of the locomotion test. The center area, defined as a square of 20 × 20 cm, was specifically designated for this measurement.

### Prepulse inhibition test

Prepulse inhibition (PPI) was assessed using the SR-LAB Startle Response system (San Diego Instrument, USA). Mice were placed in plexiglass tubes for the duration of the test. Each test session began with a 5-min acclimation period at a background noise level of 65 dB. During the test, the mice underwent three types of trials: background noise only (65 dB, 40 ms in duration), pulse alone (120 dB, 40 ms in duration), and a prepulse pulse with a 120-dB stimulus (40 ms in duration) preceded by a 20-ms prepulse at 78, 82, 86, and 90 dB, with a 100-ms interval between the prepulse and pulse. Trials were presented randomly with a variable intertrial interval (ITI) ranging from 10 to 20 s. Each trial type was repeated six times. The percentage of PPI was calculated as follows: PPI = (startle amplitude of pulse alone - startle amplitude of prepulse pulse)/startle amplitude of pulse alone × 100%.

### Forced swim test

The forced swim test (FST) is a highly reliable test for evaluating depressive-like behavioral states. Mice were placed individually in a vertical transparent cylinder (40 cm in height and 15 cm in diameter) containing tap water at 23 ± 1 °C and 20 cm in depth. The time of immobility was defined as the immobile time spent by the mice floating in the water with no active movements but movements that were necessary to keep their heads above the water. After 2 min of habituation, the immobility time of the mice was recorded for an additional 4 min. The immobility time was recorded and analyzed by the EthoVision XT video tracking software.

### Brain slice preparation

Mice of both sexes aged between 4 and 9 weeks were deeply anesthetized using pentobarbital (intraperitoneally) and then transcardially perfused with ice-cold partial sucrose solution (PSS) containing the following (mM): 80 NaCl, 2.5 KCl, 24 NaHCO_3_, 1.25 NaH_2_PO_4_, 25 glucose, 75 sucrose, 0.5 CaCl_2_, 4 MgCl_2_, 1 ascorbic acid, and 3 sodium pyruvate saturated with 95% O_2_/5% CO_2_. The brain was glued onto the stage of a vibrating microtome (Lecia VT 1200S, Germany). Coronal brain sections (300 μM) containing the dmPFC were cut in ice-cold oxygenated cutting solution, and the slices were subsequently transferred to a storage chamber lined with mesh containing regular ACSF consisting of in (mM) 125 NaCl, 2.5 KCl, 25 NaHCO_3_, 1.25 NaH2PO_4_, 30 glucose, 2 CaCl_2_, and 1 MgCl_2_ at 34 °C for 30 min. Subsequently, the slices were incubated at room temperature for an additional 1 h before recording.

### Immunofluorescence staining

Coronal brain slices from 8-week-old mice (300 μm thick) were prepared (see above). The slices were subsequently divided into control and drug treatment groups. Control slices were incubated with the vehicle control in ACSF, while drug treatment slices were incubated in ACSF containing 10 μM MPX-004 for 90 min at room temperature; all slices were incubated in 95% O_2_ and 5% CO_2_. Following incubation, the slices were fixed in 4% paraformaldehyde (PFA) for 12 h and then immersed in 30% sucrose solution for 24 h at 4 °C. The slices were sectioned at 40 μm using a cryomicrotome and collected in 0.01 M phosphate-buffered saline (PBS).

Brain sections were washed three times with 0.01 M phosphate-buffered saline (PBS) for 10 min each at room temperature. The sections were then incubated in a blocking solution for 2 h at room temperature. The blocking solution consisted of 0.01 M PBS containing 10% normal goat serum and 0.3% Triton X-100 to minimize nonspecific binding. Following blocking, the brain sections from C57BL6J mice were incubated with the following primary antibodies: mouse anti-CaMKII (1:200; MA1–048; lot XL365953; Invitrogen, USA) and guinea pig anti-c-Fos (1:350,000; 226308; Synaptic Systems) diluted in 0.01 M PBS containing 0.3% Triton X-100 (PBST). This incubation was carried out for 18 h at 4 °C. The brain sections were washed three times with 0.01 M PBS for 10 min at room temperature. The sections were then incubated with the following secondary antibodies: goat anti-mouse Alexa Fluor 488 (1:500; ab150113; lot 1001014621, Abcam, UK) and goat anti-guinea Pig Alexa Fluor 594 (1:500; A11076; lot 2540867, Invitrogen, USA) diluted in 0.01 M PBS for 2 h at room temperature. For PV Cre::Ai14 mice, sections were incubated with the following primary antibodies: guinea pig anti-c-Fos (1:350,000; 226308, Synaptic Systems, Germany) and goat anti-RFP (1:2000; 200–101-379; lot 50005; Rockland Immunochemicals, USA). The secondary antibodies used were donkey anti-guinea pig Alexa Fluor 488 (1:500; 706–545-148, Jackson ImmunoResearch, USA) and donkey anti-goat Cy3 (1:500; 705–165-003, Jackson ImmunoResearch, USA). For SST Cre::Rosa-YFP mice, sections were first incubated with the following primary antibodies: chicken anti-GFP (1:2000; GFP-1020; lot GFP3717982; Aveslab, USA) and guinea pig anti-c-Fos (1:350,000; 226308; Synaptic Systems, Germany). The secondary antibodies used were goat anti-chicken Alexa Fluor 488 (1:500; A-11039, Thermo, USA) and goat anti-guinea Pig Alexa Fluor 594 (1:500; A11076, Invitrogen, USA). After three additional washes in 0.01 M PBS, the sections were incubated with DAPI (100 ng/mL) in 0.01 M PBS for 8 min. The sections were then washed three more times in 0.01 M PBS for 10 min each at room temperature. Finally, the sections were mounted on glass slides, covered with coverslips, and imaged using a Nikon confocal microscope with a 10 × lens. Image analysis and quantification were performed using Imaris software.

### Whole-cell recordings using brain slices

For spontaneous excitatory postsynaptic currents (sEPSCs) and spontaneous inhibitory postsynaptic currents (sIPSCs), brain slices were incubated in ACSF containing either vehicle control (0.02% DMSO) or MPX-004 (10 μM) for 20 min. After incubation, the slices were transferred to a recording chamber and continuously perfused with oxygenated ACSF at 33 °C and a flow rate of 2 to 4 ml/min. Layer V PYNs were visualized using an infrared camera. PV-INs were identified by red fluorescence from PV::Ai14, whereas SST-INs were identified by green fluorescence from SST::Rosa-YFP. Glass pipettes, with a resistance of 3–6 MΩ, were pulled using a micropipette puller (PC-100, Narishige, Japan). Whole-cell recordings were performed using a HEKA amplifier (EPC-10 USB, Germany). Data were filtered at 8 kHz, digitized at 20 kHz, and acquired using PatchMaster software on a PC.

For sEPSC recordings, PYNs were held at −77 mV using an internal solution containing (in mM) 135 K gluconate, 5 KCl, 0.1 EGTA, 10 HEPES, 2 NaCl, 5 Mg-ATP, 0.4 Na_2_-GTP, and 10 Na_2_-phosphocreatine, adjusted to pH 7.2–7.3 with KOH, with an osmolarity of 290–295 mOsm. The Cl^−^ reversal potential was experimentally determined to be −77 mV.

For sIPSCs and NMDA receptor-mediated evoked postsynaptic currents (NMDAR-EPSCs), the internal solution contained (in mM) 130 CsMeSO_3_, 4 NaCl, 4 Mg-ATP, 0.3 Na_2_-GTP, 0.5 EGTA, 10 HEPES, and 5 QX-314Cl, adjusted to pH 7.2–7.3 with CsOH, with an osmolarity of 290–295 mOsm. sIPSCs were recorded at 0 mV to avoid interference from AMPA and NMDA receptor currents. NMDAR-EPSCs were recorded at +40 mV in the presence of 10 μM DNQX to block non-NMDA ionotropic glutamate receptors and 10 μM gabazine or 50 μM picrotoxin to block GABA_A_ receptors. NMDAR-EPSCs were elicited by current pulses of 50–300 μA 0.1 ms in duration delivered every 30 s using a concentric bipolar stimulating electrode positioned in layers II/III of the dmPFC. A stable baseline of NMDAR-EPSCs was recorded for 5–15 min, followed by 10–15 min of recording with 10 μM MPX-004 in the bath perfusion. At the end of the recordings, 50 μM DL-APV was added to confirm that the recorded NMDAR-EPSCs were mediated by NMDA receptors. During sEPSC, sIPSC and NMDAR-EPSC recordings, access resistance was monitored every 30 s by applying a voltage step of −5 mV for 200 ms.

To examine evoked excitatory postsynaptic potential (eEPSP)-induced action potentials in parvalbumin-expressing interneurons (PV-INs), somatostatin-expressing interneurons (SST-INs), and pyramidal neurons (PYNs), the cells were held at −65 mV in current-clamp mode. A train of 10 electrical pulses at 20 Hz with a stimulation intensity of 80 μA was delivered. This protocol typically elicits one or more action potentials in PV-INs while inducing one or no action potentials in SST-INs and PYNs. To assess the effects of MPX-004 on eEPSP-induced spiking, the same pulse train (10 pulses at 20 Hz) was applied before and after bath application of MPX-004 (10 μM) for at least 6 min.

Data from neurons with an initial access resistance greater than 20 MΩ or those exhibiting a change of 20% or more in access resistance during the recordings were excluded.

### Cell type-specific knockdown of GluN2A using a cre-dependent AAV-shRNA system in PV-cre mice

We used an AAV vector to knock down Grin2a expression (Sunbio Medical Biotechnology Company, Shanghai, China; AAV-EF1A-EGFP-U6-*shGrin2A*, 1.39 × 10^13^ vg/mL; AAV-EF1A-EGFP-U6-control, 1.86 × 10^13^ vg/mL), and its knockdown efficiency was validated using qPCR and Western blotting (see Supplemental Fig. 3A–E). The shRNA targeted the Grin2a gene (RefSeq NM_008170; CDS nucleotides 1743–1763), with a sense sequence of 5′-GCACCAGTACATGACCAAATT-3′, an antisense sequence of 5′-AATTTGGTCATGTACTGGTGC-3′, and a loop region of CTCGAGA. Specificity was confirmed by querying the 21-nt antisense sequence against the *Mus musculus* transcriptome with NCBI BLAST (blastn-short; RefSeq_RNA; taxid 10090; word size 7; E value 1000; low-complexity masking OFF). No transcripts other than Grin2a showed ≥17-nt contiguous identity, and no 7-mer seed matches were found in 3’UTRs, indicating the absence of high-confidence off-targets (page 13). Using the same shRNA sequence, we subsequently constructed a Cre-dependent version of the virus (AAV-EF1A-Dio-EGFP-U6-*shGrin2A*, 1.03 × 10^13^ vg/mL; AAV-EF1A-Dio-EGFP-U6-control, 1.24 × 10^13^ vg/mL) to achieve cell type-specific knockdown of GluN2A in PV-Cre mice.

### RNA extraction and RT-qPCR analysis of mPFC tissue

Medial prefrontal cortex (mPFC) tissue was dissected and homogenized for mRNA quantification. The homogenate was centrifuged at 12,000 × *g* for 15 min at 4 °C, and the supernatant was collected. Total RNA was extracted using the SteadyPure Quick RNA Extraction Kit (AG21023, AG, China). Complementary DNA (cDNA) was synthesized from the extracted RNA using the Evo M-MLV RT Mix Kit with gDNA Clean for qPCR Ver.2 (AG11728, AG, China). Quantitative real-time PCR (RT-qPCR) was performed using the SYBR Green Premix Pro Taq HS qPCR Kit (AG11702, AG, China) on a real-time PCR system (ABI7500, Applied Biosystems, USA).

The relative mRNA expression levels of *Grin2a, Grin2b* and *Grin1* in the experimental and control groups were calculated using the 2^−ΔΔCT^ method, with GAPDH used for normalization (see Supplemental Table 2 for the primer list).

### Western blot analysis of mPFC protein expression

The medial prefrontal cortex (mPFC) was dissected and homogenized at a ratio of 100:1:1 in RIPA lysis buffer (P0013B, Beyotime, China), a protease inhibitor cocktail for general use, 100X (P1005–1, Beyotime, China) and 0.1 M EDTA (P1005–2, Beyotime, China). The homogenate was centrifuged at 12,000 × *g* for 5 min at 4 °C, and the supernatant was collected for protein extraction. Protein quantification was performed using a BCA protein assay kit (23225, Thermo Fisher Scientific, USA) according to the manufacturer’s instructions. Equal amounts of protein (40 μg per lane) were separated by SDS-PAGE with a lower gel percentage of 7.5% and transferred to a PVDF membrane.

The membrane was blocked with 5% nonfat milk in TBST for 2 h at room temperature, followed by incubation with the following primary antibodies overnight at 4 °C (16–18 h): mouse monoclonal anti-β-actin antibody as a loading control (1:20,000, RM2001, Beijing Ray Antibody Biotech, China) or rabbit monoclonal anti-GluN2A antibody (1:500, A6473, Invitrogen, USA). After being washed 3 × 10 min in TBST, the membrane was incubated with the following HRP-conjugated secondary antibodies for 2 h at room temperature: goat anti-mouse IgG (H + L)-HRP (1:5,000, Beijing Ray Antibody Biotech, China) or goat anti-rabbit IgG (H + L)-HRP (1:5,000, C31460100, Invitrogen, USA). Protein bands were visualized using BeyoECL Star (P0018AS, Beyotime, China) and imaged with a Bio-Rad Universal Hood II system (Tannon, USA). Quantitative analysis was performed via ImageJ software (NIH, USA). Band intensities were normalized to that of β-actin, and relative protein expression levels were calculated.

### Public scRNA-seq database analysis

scRNA-seq datasets (GSM6422992, GSM6422993, and GSM6422994) were downloaded from the Gene Expression Omnibus (www.ncbi.nlm.nih.gov/geo). The datasets were processed using Seurat (v.5.1.0) in R (v.4.4.1). Standard analysis was performed as follows: cells with a mitochondrial gene ratio >20% were filtered out. Then, 8357 excitatory neurons (PYN) were annotated by *Slc17a6* and *Slc17a7* as markers. Inhibitory neurons were annotated by *Gad1* and *Gad2* as markers. The filtered dataset was further divided into 102 *Pvalb*-positive interneurons (PV-INs), 465 *Sst*-positive interneurons (SST-INs) and other interneurons. Only the PV-IN, SST-IN and PYN data were extracted for DEG analysis.

### Chemical reagents

*D*,*L*-2-amino-5-phosphonovalerate (DL-APV, ab120004, Abcam, USA) and 7-chlorokynurenic acid (7-CKA, ab120024, Abcam, USA) were purchased from Abcam. Gabazine (SR95531, HY-103533, MCE, USA), picrotoxin (PTX, HY-101391, MCE, USA) and dimethylsulfoxide (DMSO, HY-Y0320, MCE, USA) were obtained from MedChemExpress. MPX-004 (M-280) was purchased from Alomone Labs. All the other chemical reagents were purchased from Sigma-Aldrich.

### Data and statistical analysis

Patch-clamp data from mouse brain slices were analyzed using Axograph (Axograph Scientific, Sydney, Australia). For event detection of spontaneous excitatory postsynaptic currents (sEPSCs), spontaneous inhibitory postsynaptic currents (sIPSCs) and spontaneous postsynaptic potentials (PSPs), raw data were digitally filtered at 1 kHz. The optimal detection threshold was set by calculating the nearest 0.5 increment between 3 and 4.5 times the standard deviation of noise, ensuring a false-positive rate below 0.05. Cells with a false-positive/positive ratio greater than 5.5 were excluded from further analysis. The deactivation time courses of the NMDA-EPSCs were analyzed using Axograph and fitted using the following two-exponential function: *I*_total_ = *I*_fast_ exp (-time/τ_fast_) + *I*_slow_ exp (-time/τ_slow_), where τ_fast_ and τ_slow_ represent the deactivation time constants for the fast and slow components, respectively, and τ_fast_ and τ_slow_ are the corresponding current amplitudes. The weighted deactivation time constant was calculated as follows: τ_weighted_ = (τ_fast_*I*_fast_ + τ_slow_*I*_slow_)/(*I*_fast_+ *I*_slow_).

Statistical analyses were conducted using GraphPad Prism. Comparisons between two groups were performed using unpaired *t* tests, paired *t* tests, Mann-Whitney tests or Wilcoxon matched-pairs signed-rank tests, depending on the results of the normality test. For multiple comparisons, one-way ANOVA and two-way ANOVA with post hoc multiple comparisons were utilized. The data are presented as the means ± SEMs. The notation “ns” indicates no significant difference. Significance levels were set at *P < 0.05, **P < 0.01, ***P < 0.001 and ****P < 0.0001. The full descriptions of the means ± SEMs, group sizes and statistical tests are provided in the supplemental tables.

## RESULTS

### MPX-004 is a potent and selective inhibitor of GluN2A-containing NMDA receptors

TCN-201, a prototypical negative allosteric modulator of the GluN2A subunit, has limited solubility (up to 5 μM in saline), and its efficacy in inhibiting GluN2A-containing NMDA receptors is highly sensitive to glycine concentrations because TCN-201 inhibits GluN1/2A NMDA receptors by reducing glycine binding at the GluN1 site [54]. In contrast, MPX-004, an analog of TCN-201, shows improved solubility (up to 10 μM in saline) and reduced sensitivity to glycine concentrations during the tonic activation of GluN2A-containing NMDA receptors [46]. While both TCN-201 at 5 μM and MPX-004 at 10 μM show selective inhibition of tonically activated diheteromeric GluN1/2 A NMDA receptors without affecting GluN1/2B, GluN2C, or GluN2D NMDA receptors (for detailed information, see Supplemental Fig. 1 in Yi et al. [46]), their inhibitory selectivity and efficacy remain uncertain during brief NMDA receptor activation.

**Fig. 1:**
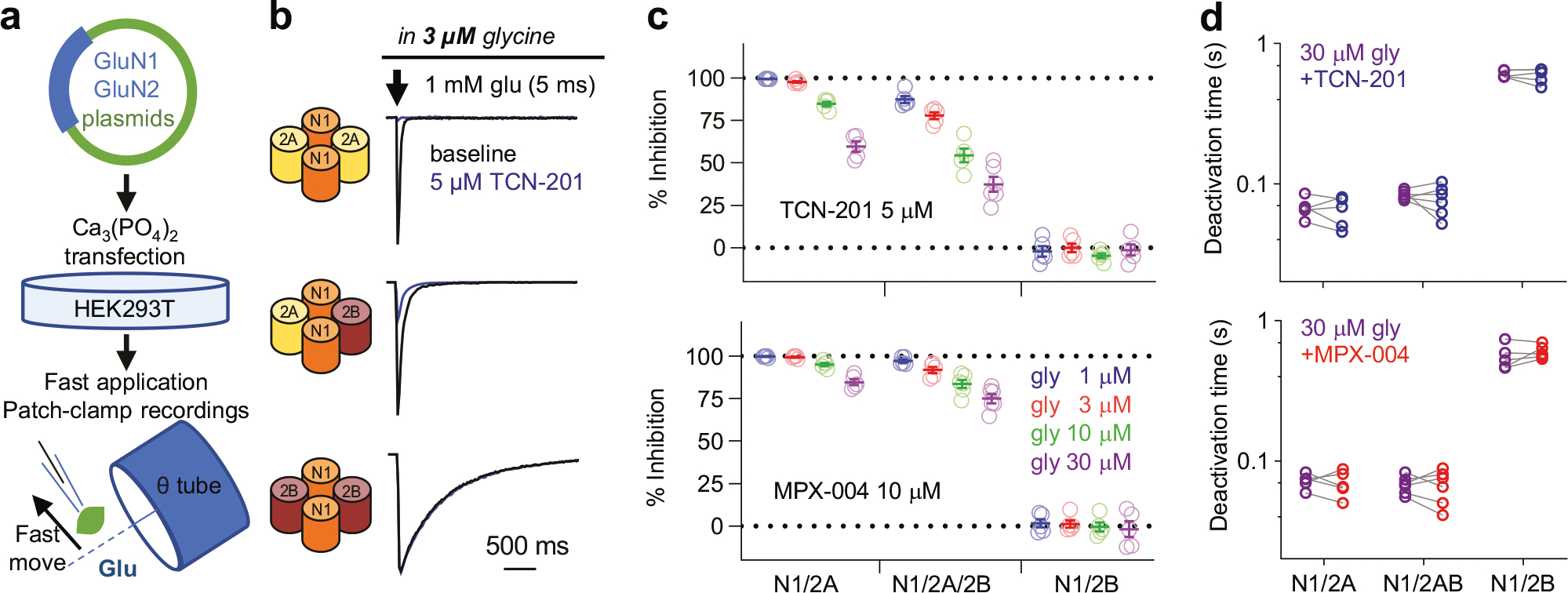
Inhibitory efficacy and selectivity of MPX-004 and TCN-201 on NMDA receptor subtypes. **a** Schematic diagram showing the expression of various NMDA receptor subtypes in HEK293T cells. NMDA receptor-mediated currents were activated by brief (5 ms) exposure to 1 mM glutamate in the continuous presence of glycine at concentrations of 1, 3, 10, or 30 μM. **b, c** TCN-201 treatment resulted in complete blockade of currents mediated by GluN1/2 A receptors, partial blockade of currents mediated by GluN1/2 A/2B receptors, and no effect on currents mediated by GluN1/2B receptors. **c** MPX-004 treatment completely blocked currents mediated by both GluN1/2 A and GluN1/2 A/2B but did not affect currents mediated by GluN1/2B receptors. **d** TCN and MPX-004 did not affect the deactivation time constant of GluN1/2 A, GluN1/2 A/2B or GluN1/2B NMDA receptors (see Supplemental Table 3 for complete statistical information).

To investigate this, we expressed recombinant NMDA receptors in HEK293T cells (Fig. 1a) and evaluated the effects of TCN-201 and MPX-004 on diheteromeric GluN1/2 A and GluN1/2B, as well as triheteromeric GluN1/2 A/B NMDA receptors, using brief (5 ms) activation by 1 mM glutamate at various glycine concentrations (1, 3, 10, and 30 μM). TCN-201 at 5 μM significantly inhibited GluN1/2 A currents, with inhibition reaching 59.6% at 30 μM glycine. Notably, nearly complete blockade ( > 80%) occurred at lower glycine concentrations (1–10 μM, Fig. 1b, c). For GluN1/2 A/2B receptors, TCN-201 achieved less than 80% blockade across 3–30 μM glycine (Fig. 1b, c). Given that brain glycine concentrations typically remain below 10 μM [55, 56], the effectiveness of TCN-201 in blocking GluN1/2 A/2B receptors is limited. In contrast, MPX-004 at 10 μM exhibited superior inhibitory effects, resulting in over 80% inhibition of both GluN1/2 A and GluN1/2 A/2B responses at physiologically relevant glycine concentrations (1–10 μM, Fig. 1c) [55]. Notably, neither TCN-201 nor MPX-004 altered GluN1/2B currents, confirming their selectivity for GluN2A-containing NMDA receptors (Fig. 1b, c). Additionally, MPX-004 did not alter the deactivation time of the NMDA receptor subtypes (Fig. 1d). Based on these findings, we selected 10 μM MPX-004 for further investigations in mice.

### MPX-004 infusion in the dmPFC impairs sensorimotor gating and reduces immobility in the forced swim test

Previous studies have consistently shown reduced functional connectivity between the prefrontal cortex and other brain regions in patients with depression [1–3, 48] and schizophrenia [2, 8]. This weakened connectivity may be attributed to the chronic nature of these diseases. In line with this speculation, elevated prefrontal connectivity has been revealed in early-stage unmedicated schizophrenia patients [44]. NMDA receptor hypofunction reduces the activity of GABAergic interneurons and enhances the activity of pyramidal cells in the mPFC [22, 57]. Recent analyses have identified *GRIN2A* as a gene associated with susceptibility to schizophrenia [39, 40], and the GluN2A subunit is also suggested to mediate the antidepressant effects of ketamine [36, 37]. However, how acute pharmacological inhibition of GluN2A regulates the microcircuit of the mPFC and related behaviors is still unclear.

**Fig. 2:**
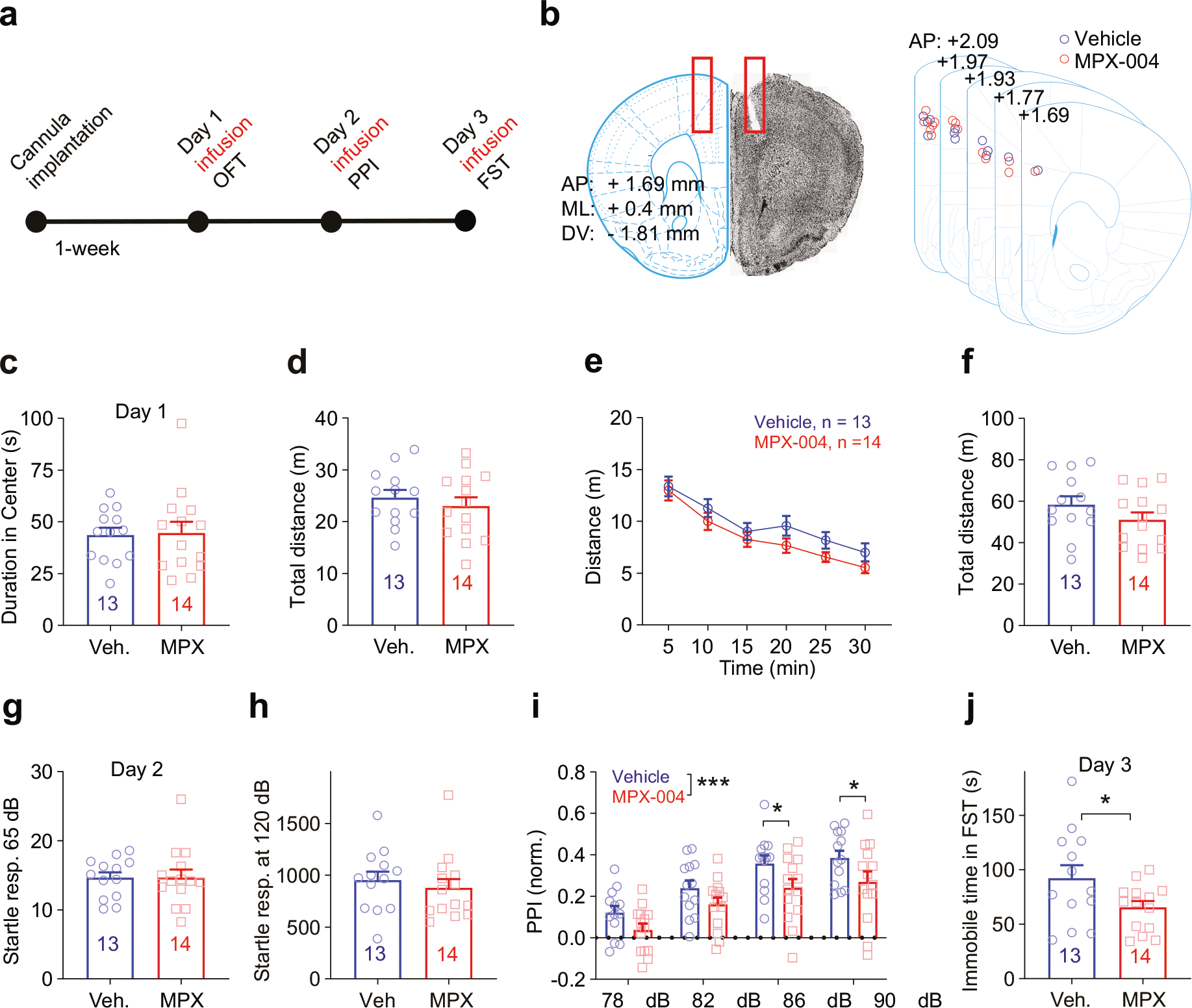
MPX-004 infusion in the dmPFC impairs sensorimotor gating and reduces immobility duration in the forced swim test. **a** Schematic diagram illustrating the guide cannula implantation procedure in the dmPFC, followed by vehicle or MPX-004 infusion and subsequent behavioral tests. **b** An image displaying the implantation coordinates of the guide cannula, accompanied by a schematic illustrating the infusion site position, confirmed through post hoc verification. **c** Time spent in the center of the open field (10 min). **d** Traveled distance (10 min). **e, f** Time course of traveled distance during the locomotion test (30 min). **g, h** Bar graphs showing no difference in the startle response measured at 65 dB or 120 dB among the vehicle control and treatment groups. **i** Bar graph demonstrating a significant reduction in prepulse inhibition in the MPX-004 (10 μM) treatment group compared with the vehicle control group. The data were analyzed by two-way ANOVA followed by multiple comparisons using the two-stage linear step-up procedure of Benjamini, Krieger and Yekutieli, *P < 0.05, ***P < 0.001. (**j**) Immobility time during the forced swim test, showing a significant reduction in immobility when the animals were treated with MPX-004. The data were analyzed by unpaired *t* test, *P < 0.05 (see Supplemental Table 4 for complete statistical information).

To explore the acute and subchronic effects of GluN2A-containing NMDA receptor blockade on behavior, we targeted the dorsomedial prefrontal cortex (dmPFC), a region implicated in both depression [1] and schizophrenia [5, 6, 22, 58, 59]. We implanted a guide cannula into the dmPFC of the right hemisphere of the brain (Fig. 2a, b) and administered either a vehicle control or MPX-004 at 10 μM. The mice were then infused with the respective solutions for three consecutive days, and behavioral tests were conducted 30 min after infusion to assess potential antianxiety effects, deficits in sensorimotor gating, and antidepressant-like effects.

On day 1, we evaluated locomotion and anxiety levels using the open field test. The mice were observed for 30 min, and the duration in the center of the field was analyzed for the first 10 min to gauge anxiety. Analysis revealed no significant differences in center dwell time or distance traveled between the vehicle and MPX-004 groups for the first 10 min (Fig. 2c, d; unpaired *t* test, *n* = 13 and 14, respectively) or for the entire 30 min (Fig. 2e, f; multiple *t* test and unpaired *t* test).

On day 2, we assessed prepulse inhibition (PPI) of the startle response. Although no significant differences were observed in startle amplitudes to stimulation at 65 dB or 120 dB (Fig. 2g, h; unpaired *t* test, *n* = 13 and 14), MPX-004 treatment resulted in a significant reduction in PPI (Fig. 2i; *P* < 0.05, two-way ANOVA, *n* = 13 and 14, respectively). These findings suggest that acute blockade of GluN2A-containing NMDA receptors impairs sensorimotor gating, a behavior associated with schizophrenia-like deficits.

On day 3, we performed the forced swim test (FST) to evaluate the potential acute antidepressant-like effects of MPX-004. The results revealed a significant reduction in immobility time during the FST, indicating a noteworthy antidepressant-like effect (Fig. 2j; *P* < 0.05, unpaired *t* test).

### MPX-004 locally reduces PV-interneuron activity and enhances pyramidal neuron activity in the dmPFC

**Fig. 3:**
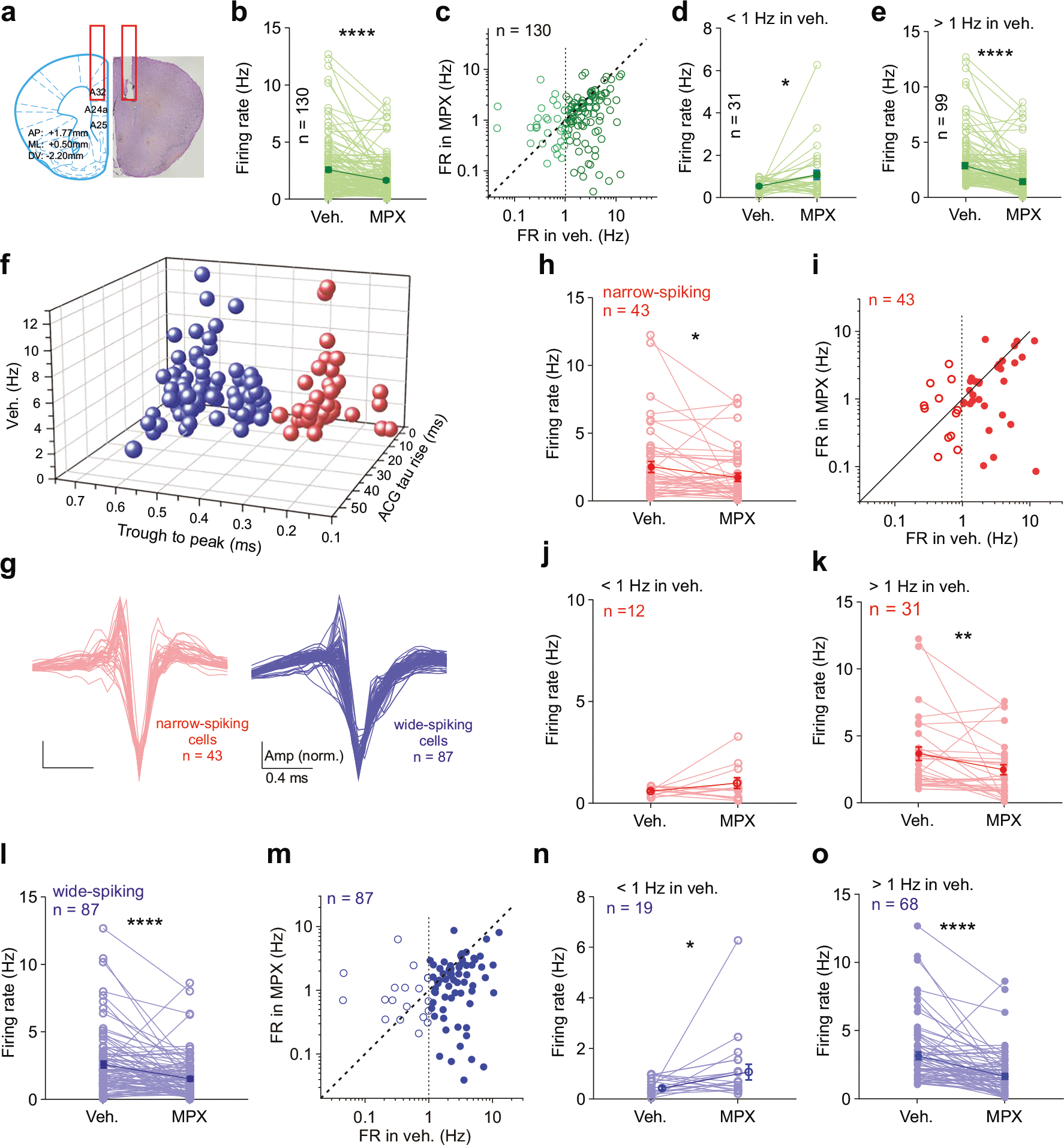
Local MPX-004 infusion regulates dmPFC neuronal activity. **a** Schematic illustration of tetrode recording in the dmPFC with a cannula for drug administration. **b** Bar graph showing that MPX-004 suppresses the overall firing rate of recorded neurons (2.72 ± 0.22 vs. 1.76 ± 0.15 Hz, Wilcoxon matched-pairs signed-rank test, ****P < 0.0001). **c** Scatter plot showing the firing rates (FRs) of recorded cells in the vehicle and MPX-004 groups. **d** Bar graph showing that MPX-004 enhances the firing rates of recorded neurons with basal firing rates lower than 1 Hz (0.55 ± 0.05 vs. 1.10 ± 0.22 Hz, Wilcoxon matched-pairs signed-rank test, *P = 0.017). **e** Bar graph showing that MPX-004 reduces the firing rates of recorded neurons with basal firing rates higher than 1 Hz (3.39 ± 0.25 vs. 1.96 ± 0.18 Hz, Wilcoxon matched-pairs signed-rank test, ****P < 0.0001). **f** Classification of a total of 130 recorded cells from six mice based on the trough-to-peak time (0.4 ms). **g** Z-scored waveforms of narrow-spiking (red) and wide-spiking neurons (blue). **h, l** MPX-004 significantly reduces the overall firing rates of both narrow-(2.7 ± 0.42 vs. 1.9 ± 0.30 Hz, Wilcoxon matched-pairs signed-rank test, *P = 0.033) and wide-spiking neurons (2.72 ± 0.26 vs. 1.66 ± 0.17 Hz, Wilcoxon matched-pairs signed-rank test, ****P < 0.0001) in the dmPFC. **i, m** The effects of MPX-004 on the neuron firing rate are activity dependent. **j, n** When the basal firing rate is less than 1 Hz, MPX-004 enhances the firing rate of wide-spiking neurons (0.54 ± 0.08 vs. 1.18 ± 0.32 Hz, Wilcoxon matched-pairs signed-rank test, *P = 0.036) but not narrow-spiking neurons (0.57 ± 0.06 vs. 0.97 ± 0.27 Hz, Wilcoxon matched-pairs signed-rank test, *P* = 0.27). **k, o** Neurons with basal firing rates above 1 Hz show reduced activity in response to MPX-004 in both narrow-spiking (3.54 ± 0.51 vs. 2.32 ± 0.39 Hz, Wilcoxon matched-pairs signed-rank test, **P = 0.003) and wide-spiking neurons (3.33 ± 0.29 vs. 1.80 ± 0.20 Hz, Wilcoxon matched-pairs signed-rank test, ****P < 0.0001). See Supplemental Table 5 for complete statistical information.

To investigate the neural dynamics underlying the effects of MPX-004 on specific cell populations within the dmPFC, we performed in vivo tetrode recordings (Fig. 3a); these high-resolution, multiunit recordings enabled the differentiation of cell populations by their firing properties. A total of 130 neurons from six mice were recorded. While MPX-004 infusion at 10 μM generally suppressed overall firing rates (Fig. 3b), neurons with a basal firing frequency below 1 Hz presented an increase in firing rate after treatment (Fig. 3c, d), whereas those with basal firing above 1 Hz were inhibited (Fig. 3c, e). The recorded cells were then classified as narrow-spiking (*n* = 43) or wide-spiking (*n* = 87) neurons based on their waveform properties (Fig. 3f, g). MPX-004 reduced firing rates in both classes (Fig. 3h, k, l, o). However, interestingly, it enhanced firing in wide-spiking neurons with a basal firing rate <1 Hz (Fig. 3n) without affecting narrow-spiking neurons (Fig. 3j). Our previous results of in vivo whole-cell patch-clamp recordings [11] demonstrated that the majority of putative pyramidal neurons in the prefrontal cortex (approximately 85%) exhibit firing rates below 1 Hz (Supplementary Fig. 1a–c). However, chemogenetic inactivation of parvalbumin-positive interneurons (PV-INs), but not somatostatin-positive interneurons (SST-INs), significantly increased the firing rate of pyramidal neurons (Supplementary Fig. 1a–c) without changing the mean membrane potential (Supplementary Fig. 1d, e). Together, these findings suggest that MPX-004 may increase the activity of pyramidal neurons by dampening the firing of inhibitory interneurons.

**Fig. 4:**
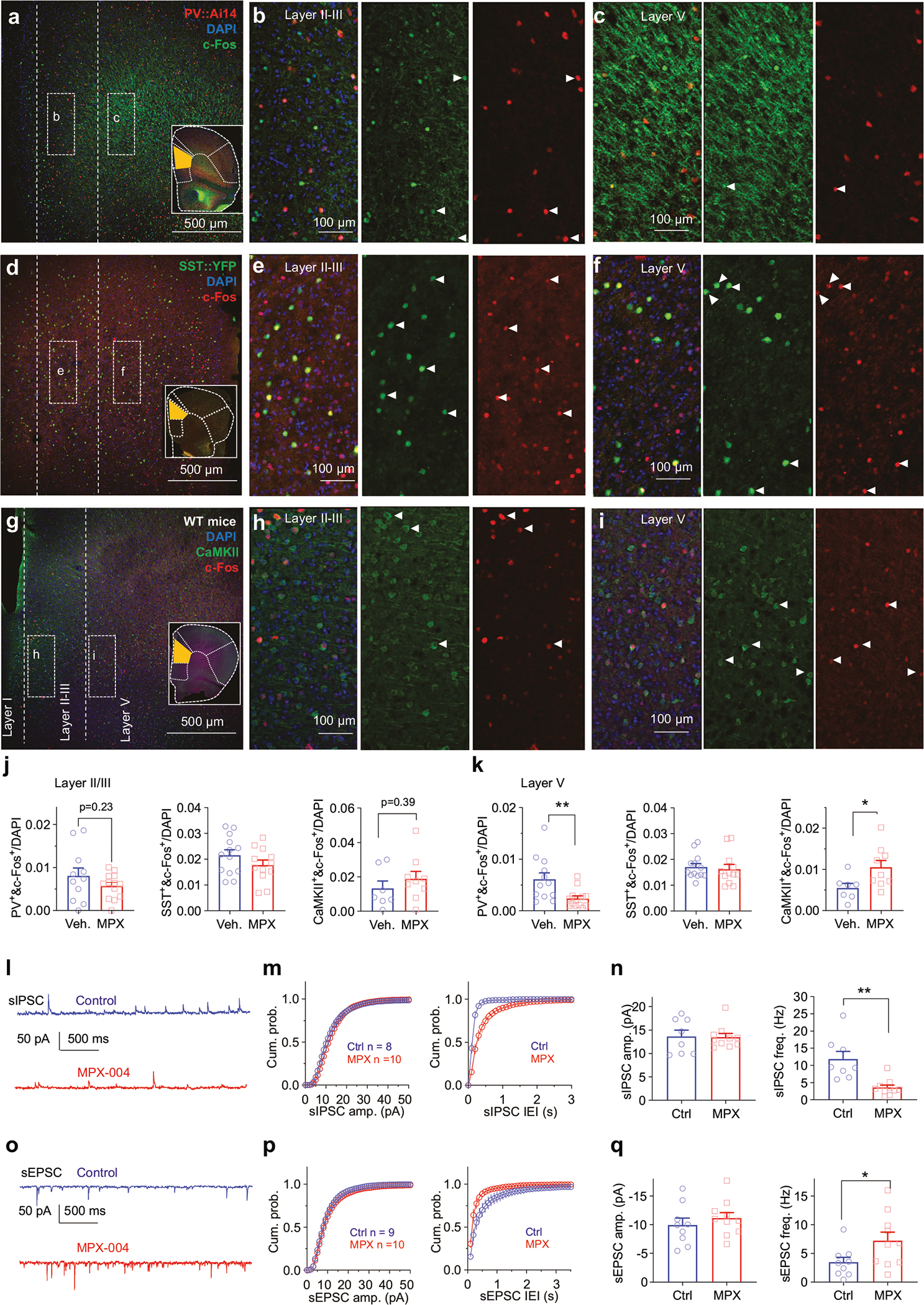
MPX-004 reduces PV-IN activity and enhances PYN activity in the dmPFC. **a** Fluorescence images of the dmPFC showing staining for c-Fos (green), PV::Ai14 (red), and DAPI (blue), with zoomed-in regions of layers II/III **b** and V **c**. The white arrows indicate cells costained with anti-c-Fos and anti-Ai14 antibodies. **d** Fluorescence images of the dmPFC showing staining for c-Fos (red), SST::YFP (green), and DAPI (blue), with zoomed-in regions of layers II/III **e** and V **f**. The white arrows indicate cells costained with anti-c-Fos and anti-GFP antibodies. **g** Fluorescence images of the dmPFC showing staining for c-Fos (red), CaMKII (green), and DAPI (blue), with zoomed-in regions of layers II/III **h** and V **i**. The white arrows indicate cells costained with anti-c-Fos and anti-CaMKII antibodies. **j** In layer II/III of the dmPFC, MPX-004 treatment did not significantly alter the percentage of c-Fos^+^ PYNs in WT mice, the percentage of c-Fos^+^ PV-INs in PV::Ai14 mice or the percentage of c-Fos^+^ SST-INs in SST::YFP mice. PV-INs and SST-INs were visualized using reporter fluorescence. The data were analyzed by unpaired *t* test. **k** In layer V of the dmPFC, MPX-004 treatment led to a significant increase in the percentage of c-Fos^+^ PYNs, a decrease in the percentage of c-Fos^+^ PV-INs, and no change in the percentage of c-Fos+ SST-INs. The data were analyzed by unpaired *t* test, *P < 0.05, **P < 0.01. Each condition was assessed in 7–13 slices obtained from 3 mice **a–k. l** Representative traces of spontaneous excitatory postsynaptic currents (sIPSCs) recorded from PYNs, illustrating baseline activity and activity following the application of 10 μM MPX-004. **m** Cumulative probability plots of sIPSC amplitude showing no significant change with MPX-004 treatment. However, the cumulative probability plot of sIPSC interevent intervals exhibited a rightward shift, indicating a decreased frequency of sIPSCs. **n** Bar graph quantifying the sIPSCs showing no change in amplitude but a significant decrease in frequency with MPX-004 application. The data were analyzed by unpaired *t* test, **P < 0.01. **o** Representative traces of spontaneous inhibitory postsynaptic currents (sEPSCs) recorded from PYNs at baseline and in the presence of MPX-004. **p** Cumulative probability plots of sEPSC amplitude showing no significant change with MPX-004 treatment. The cumulative probability plot of sEPSC interevent intervals demonstrated a leftward shift, reflecting an increased frequency of sEPSCs. **q** Bar graph depicting sEPSCs showing no change in amplitude but a significant decrease in frequency following MPX-004 application. Each condition was assessed in 8–10 cells obtained from 3 mice **l–q**. See Supplemental Table 6 for complete statistical information.

Given that in vivo recording without optogenetic verification cannot unambiguously identify the cell types of recorded neurons, we performed layer-specific c-Fos staining using dmPFC brain slices incubated for 90 min with either a vehicle control or 10 μM MPX-004 (Fig. 4a–i) to further probe the effects of GluN2A inhibition on neuron types. c-Fos, an immediate-early gene, serves as a marker for neural activity [60].

PV-INs and SST-INs together account for approximately 70% of all interneurons [61]. Previous studies have indicated that chronic stress, a significant factor in the onset of depression, can increase synaptic inhibition mediated by PV-Ins [62] and reduce the expression of parvalbumin and somatostatin in postmortem tissue from schizophrenia patients [17, 18]. Thus, we aimed to examine the activity of both PV-INs and SST-INs, as well as pyramidal neurons (PYNs), in the dmPFC of control and MPX-004-treated slices.

Our findings revealed a significant decrease in the number of c-Fos^+^ PV-INs in layer V (Fig. 4a, c, k; unpaired *t* test, *P* = 0.009, *n* = 12 and 13 slices for the vehicle and MPX-004 groups, respectively) but no change in layers II/III (Fig. 4a, b, j; unpaired *t* test, *P* = 0.23, *n* = 11 and 13 slices for the vehicle and MPX-004 groups, respectively). No significant change in somatostatin-positive interneurons (SST-INs) was observed in either layer (Fig. 4d–f, j–k; unpaired *t* test, *n* = 13 and 12 slices for the vehicle and MPX-004 groups, respectively). Heterozygous PV::Ai14 and SST::YFP mice were used for the identification of PV-INs and SST-INs with florescent proteins. In contrast, MPX-004 significantly increased the number of c-Fos^+^ pyramidal neurons (PYNs) in layer V (Fig. 4g, i, k; unpaired *t* test, *P* = 0.034, *n* = 7 and 9 slices for the vehicle and MPX-004 groups, respectively) but not in layers II/III (Fig. 4g, h, j; unpaired *t* test, *P* = 0.39, *n* = 7 and 9 slices for the vehicle and MPX-004 groups, respectively).

Patch-clamp recordings of layer V PYNs in dmPFC slices further supported these findings, showing that MPX-004 treatment reduced spontaneous inhibitory postsynaptic current (sIPSC) frequency (Fig. 4l–n; *P* = 0.0013, *n* = 8 and 10 for control and MPX-004, respectively) while increasing spontaneous excitatory postsynaptic current (sEPSC) frequency (Fig. 4o–q; *P* = 0.048, *n* = 9 and 10 for control and MPX-004, respectively) without altering the amplitude of either (Fig. 4l–n, o, p).

Together with our c-Fos staining results (Fig. 4a–k), these results suggest that this increase in the E/I ratio results from the reduced activity of PV-INs and consequently disinhibition of PYNs, indicating that PV-INs are more susceptible than SST-INs to MPX-004 inhibition. However, this susceptibility of PV^+^ cells does not appear to result from higher expression levels of GluN2A, as analysis of a published cortex single-cell RNA sequencing database [63] did not support higher *Grin2a* mRNA levels in PV-INs than in SST-INs or PYNs (Supplemental Fig. 2).

**Fig. 5:**
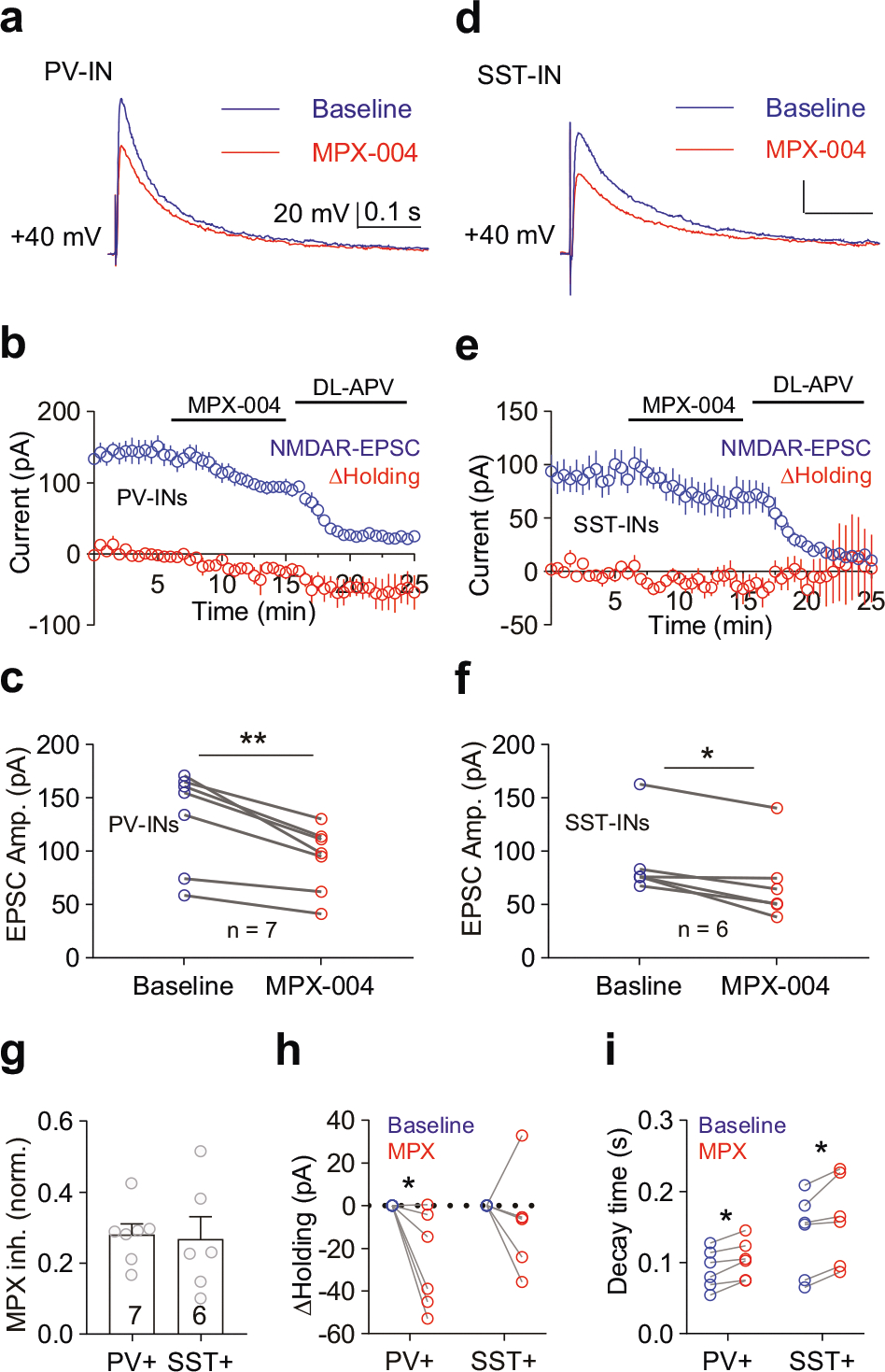
PV and SST neurons express similar GluN2A-containing NMDA receptors. Representative traces of NMDAR-EPSCs recorded from **a** PV-IN a and SST-IN **d** at +40 mV, showing baseline activity (blue) and activity in the presence of MPX-004 (red). **b, e** Time-course graphs depicting the amplitude of NMDAR-EPSCs (blue) and changes in the holding current (red) in PV-INs **b** and SST-INs **e** following treatment with 10 μM MPX-004 and 50 μM DL-APV. **c, f** Graphs showing a decrease in the amplitude of NMDAR-EPSCs in both PV-INs **c** and SST-INs **f** following MPX-004 treatment. The data were analyzed by paired *t* test **c** and Wilcoxon matched-pairs signed-rank test **f**, *P < 0.05, **P < 0.01. **g** Bar graph indicating no significant difference in the percentage of MPX-004-sensitive NMDAR-EPSCs between PV-INs and SST-INs. The data were analyzed by unpaired *t* test. **h** Holding current was significantly reduced by MPX-004 treatment in PV-INs but not in SST-INs. The data were analyzed by two-way ANOVA, followed by Sidak’s multiple-comparisons test, *P < 0.05 **i** The decay time constant of NMDAR-EPSCs was prolonged during MPX-004 treatment in both PV-INs and SST-INs. The data were analyzed by two-way ANOVA, followed by Sidak’s multiple-comparisons test, *P < 0.05. Each condition was assessed in 6–7 slices obtained from 4 mice. See Supplemental Table 7 for complete statistical information.

To investigate this mechanism, we examined the contribution of GluN2A-containing NMDA receptors to postsynaptic currents (NMDAR-EPSCs) (Fig. 5a, d). MPX-004 at 10 μM resulted in a significant reduction in NMDAR-EPSCs in both PV-INs (Fig. 5b, c, g; 28.1% ± 3.0%, paired *t* test, *P* = 0.002, *n* = 7) and SST-INs (Fig. 5e, f, g; 26.7% ± 6.3%, paired *t* test, *P* = 0.008, *n* = 6), with no significant difference between the two populations (Fig. 5g; unpaired *t* test, *n* = 7 vs. 6 for PV-INs vs. SST-INs). Consistent with the rapid deactivation kinetics of diheteromeric and triheteromeric GluN2A-containing receptors compared with diheteromeric GluN2B-containing NMDA receptors [49], the deactivation time constant was lower in the NMDAR-EPSCs of both PV-INs and SST-INs following MPX-004 treatment (Fig. 5i). Interestingly, although MPX-004 caused a similar reduction in the amplitude of NMDAR-EPSCs in both PV-INs and SST-INs, it induced holding currents in PV-INs (Fig. 5h; *P* < 0.05, paired *t* test) but not in SST-INs, suggesting tonic activation of GluN2A-containing NMDA receptors in PV-INs.

### The heightened activity state of PV-INs may underlie their high susceptibility to GluN2A inhibition

**Fig. 6:**
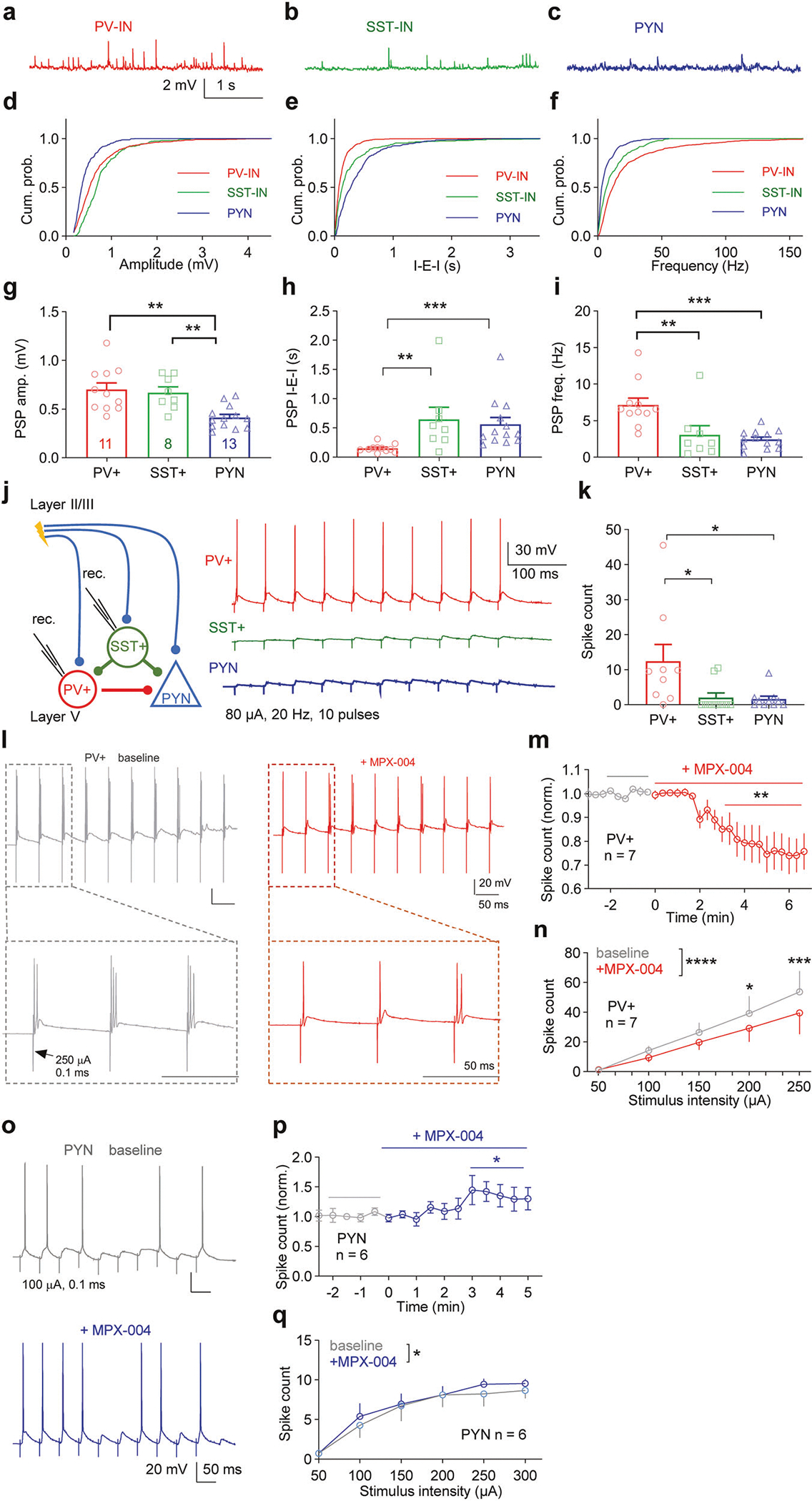
Compared with SST-INs, PV-INs receive stronger excitatory input and are more responsive. **a–c** Representative traces of spontaneous postsynaptic potentials (PSPs) recorded at the resting membrane potential from a PYN, a PV-IN, and an SST-IN in layer V of the dmPFC. **d–f** Cumulative probability distributions of the PSP amplitude, interevent interval (I-E-I), and frequency for PYNs, PV-INs, and SST-INs. **g** Bar graph showing larger PSP amplitudes in PV-INs and SST-INs than in PYNs. **h, i** Bar graphs illustrating that the PSP frequency is highest in PV-INs among PYNs, PV-INs, and SST-INs (*n* = 11, 8, and 13 from 3 PV-AI14, 3 SST-YPF and 4 WT mice, respectively). The data were analyzed by one-way ANOVA followed by Tukey’s multiple-comparisons test/Dunnett’s T3 multiple-comparisons test, *P < 0.05, **P < 0.01 for **g–i. j** Voltage responses (−65 mV) in layer V PV-INs and SST-INs following a train of stimulation (80 μA, 20 Hz, 10 pulses) delivered via a bipolar electrode positioned at layer II/III. **k** Bar graph showing that, compared with SST-INs and PYNs, PV-INs are more responsive to the same stimulation train (*P < 0.05, Kruskal-Wallis test followed by Dunn’s multiple-comparisons test, *n* = 9, 10, and 10 cells from 3 PV-AI14, 3 SST-YFP and 3 WT mice, respectively). **l** Demo traces of a typical PV-IN response to the same stimulation train before and after MPX-004 treatment. MPX-004 treatment significantly decreased the spike number induced by the pulse train with the same **m** or different stimulus intensities **n** (*n* = 7 from 3 mice). **o** Demo traces of a typical PYN response to the same stimulation train before and after MPX-004 treatment. MPX-004 treatment significantly decreased the spike number induced by the pulse train with the same **p** or different stimulus intensities **q**. Paired *t* tests **m, p** and two-way ANOVA followed by Sidak’s multiple-comparisons tests **n, q** were used to determine significant differences (*n* = 6 from 3 mice). See Supplemental Table 8 for complete statistical information. *P < 0.05, **P < 0.01, ***P < 0.001, ****P < 0.0001.

The observed tonic activation may be due to the different extents of presynaptic innervation of PV-INs and SST-INs. To investigate this, we recorded spontaneous postsynaptic potentials (PSPs) at resting membrane potentials in PV-INs and SST-INs, as well as PYNs (Fig. 6a–c). PSP amplitudes were significantly greater in PV-INs and SST-INs than in PYNs (Fig. 6g; *P* < 0.01, one-way ANOVA followed by Tukey’s multiple-comparisons test, *n* = 13, 11, and 8 for PYNs, PV-INs, and SST-INs, respectively). Notably, PSPs in PV-INs presented the highest frequency and shortest interevent interval compared with those in PYNs and SST-INs, indicating more intense presynaptic input to PV-INs (Fig. 6h, i; *P* < 0.05, one-way ANOVA).

Although PV-INs have a relatively high firing frequency in vivo, we did not observe spontaneous firing in PV-INs from prefrontal slices. This discrepancy probably results from the loss of action potential-driven presynaptic input during slice preparation. To assess the responsiveness of PV-INs, SST-INs and PYNs to action potential-driven presynaptic input, we measured their depolarization responses to a train of electrical stimuli (80 μA, 20 Hz) near their resting membrane potential (−65 mV) (Fig. 6j). Compared with SST-INs and PYNs, PV-INs generated significantly more action potentials in response to the stimulation (Fig. 6k; *P* < 0.05, one-way ANOVA), indicating that PV-INs are more responsive to presynaptic input.

As expected, the stimulus train-induced firing of PV-INs decreased upon MPX-004 application (Fig. 6l, m, *P* < 0.05, paired *t* test, *n* = 7; 6n, *P* < 0.05, two-way ANOVA). However, the stimulus train-induced firing rate of PYNs was increased by MPX-004 treatment (Fig. 6o, p, *P* < 0.05, paired *t* test, *n* = 6; 6q, *P* < 0.05, two-way ANOVA).

This finding aligns with previous in vitro and in vivo studies conducted in the mPFC. In vitro studies have shown that the frequency of sEPSCs in PV-INs is greater than that in SST-Ins [64, 65]. Additionally, in vivo data indicate that the firing rate of PV-INs surpasses that of SST-INs [66].

To further investigate the contribution of GluN2A in prefrontal PV interneurons (PV-INs) to the behavioral effects of MPX-004, we designed a Cre-dependent viral construct (AAV-DIO-shRNA-Grin2a-GFP) to enable the cell type-specific knockdown of GluN2A in PV-Cre mice (Supplemental Fig. 3f–g). The efficiency of this knockdown strategy was confirmed by recording NMDAR-mediated EPSCs in control and knockdown mice. The MPX-004-sensitive portion of the NMDAR-EPSC was significantly reduced in PV cells infected with AAV-DIO-shRNA-Grin2a-GFP compared with that in cells infected with the control virus (Supplemental Fig. 3L; *P* < 0.05, unpaired *t* test, *n* = 6 per group).

**Fig. 7:**
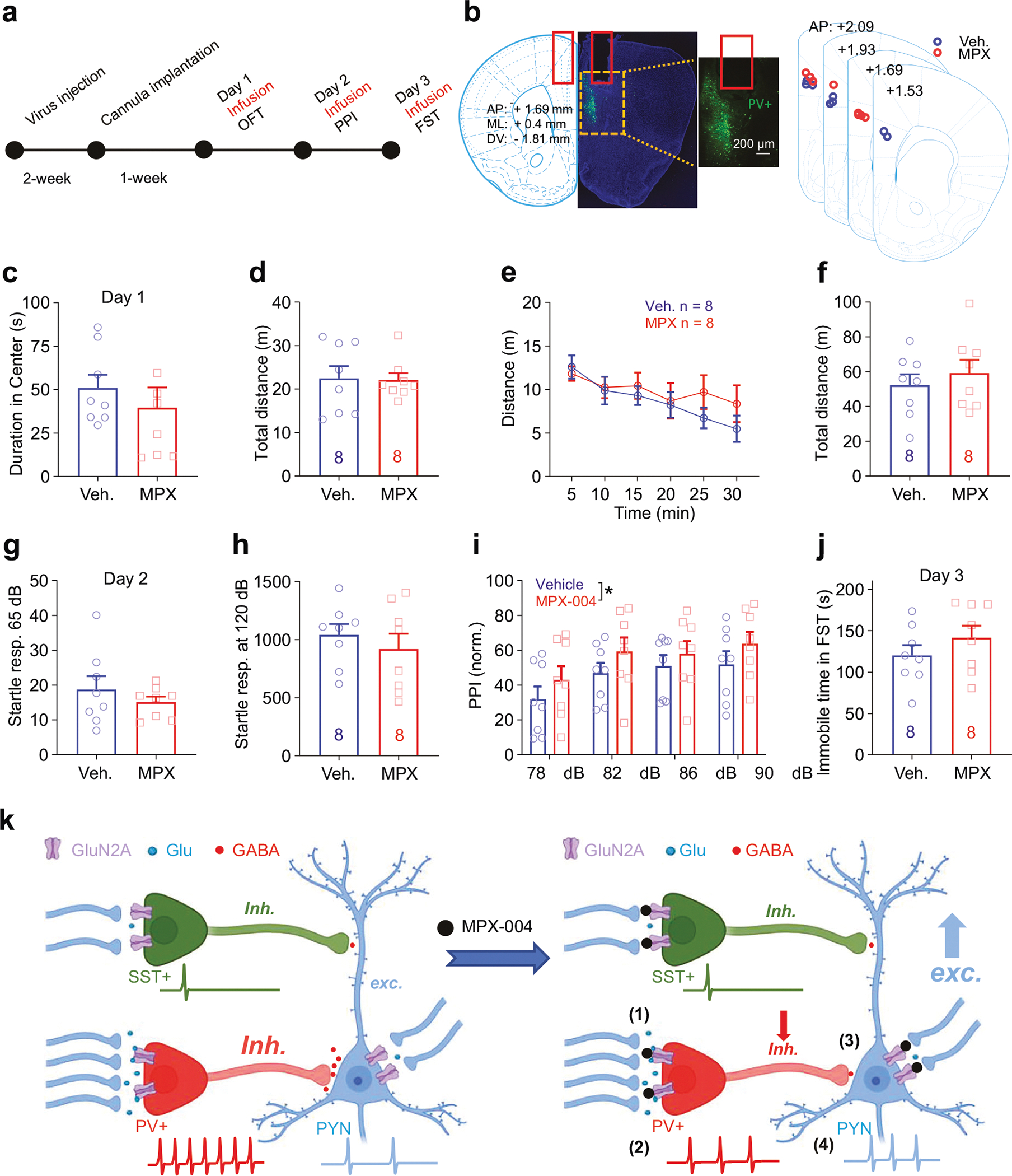
GluN2A Knockdown in prefrontal PV-INs blocks the antidepressant-like effects of MPX-004 and the deficit in sensorimotor gating. **a** Schematic diagram illustrating the viral infection and guide cannula implantation procedure in the dmPFC, followed by vehicle or MPX-004 infusion and subsequent behavioral tests. **b** An image displaying the viral infection (green) and infusion position, confirmed through *post hoc* verification. **c** Time spent in the center of the open field (10 min). **d** Traveled distance (10 min). **e, f** Time course of traveled distance during the locomotion test (30 min). **g, h** Bar graphs showing no difference in the startle response measured at 65 dB or 120 dB among the vehicle control and treatment groups. **i** Bar graph demonstrating a mildly significant increase in prepulse inhibition in the MPX-004 (10 μM) treatment group compared with the vehicle control group. The data were analyzed by two-way ANOVA followed by multiple comparisons using the two-stage linear step-up procedure of Benjamini, Krieger and Yekutieli, *P < 0.05. **j** Immobility time during the forced swim test, showing no significant change in immobility when the animals were treated with MPX-004. The data were analyzed by unpaired *t* test (see Supplemental Table 9 for complete statistical information). **k** In layer V of the dmPFC, PV-INs receive more functional excitatory presynaptic input than do SST-INs, which allows them to exert strong inhibitory control over pyramidal cell firing rates. When GluN2A-containing NMDA receptor function is impaired, such as by blockade with MPX-004 (1), PV-INs become hyperpolarized. This hyperpolarization reduces the activity of PV-INs (2), leading to diminished inhibitory control over PYNs (3) and resulting in the disinhibition of these PYNs (4).

Following viral injection to knock down GluN2A in prefrontal PV-INs (referred to as PV-Grin2a-KD) and subsequent cannula implantation, either vehicle or MPX-004 was administered prior to behavioral testing (Fig. 7a). The locations of viral expression and cannula placement were confirmed *post hoc* (Fig. 7b). MPX-004 treatment did not affect the center dwelling time or locomotor activity in the open field test (Fig. 7c–f).

Interestingly, the MPX-004-induced deficit in sensorimotor gating was abolished in PV-Grin2a-KD mice (Fig. 7g–i). In fact, MPX-004 treatment mildly enhanced sensorimotor gating in these mice (Fig. 7i; *P* < 0.05, two-way ANOVA). In the forced swim test, MPX-004 treatment did not affect immobility time in PV-Grin2a-KD mice. These findings strongly suggest that GluN2A in prefrontal PV-INs plays a key role in mediating both the antidepressant-like and pro-psychotomimetic-like effects of MPX-004.

Thus, to illustrate the impact of MPX-004 on the microcircuits of the dmPFC, we propose a working model (Fig. 7k). Given the tonic activation of GluN2A-containing NMDA receptors and the high responsiveness of PV-INs to presynaptic input, which can cause depolarization and removal of the magnesium block from NMDA receptor channels, PV-INs are particularly vulnerable to the blockade of GluN2A-containing NMDA receptors by MPX-004. This heightened sensitivity may lead to a reduced response to incoming information in PV-INs, which could subsequently cause the disinhibition of PYNs. This effect of MPX-004 on the microcircuits of the dmPFC may underlie its antidepressant-like effect and induce a sensorimotor gating deficit.

## DISCUSSION

Alterations in the GABAergic regulation of the medial prefrontal cortex (mPFC) are implicated in various brain disorders, including depression and schizophrenia [1, 5–7, 67]. The role of NMDA receptors in modulating GABAergic regulation has been extensively studied within the context of these neuropsychiatric disorders [2, 5–7, 22, 23, 31, 67]. Notably, NMDA receptor antagonists, such as ketamine, produce rapid antidepressant effects in humans [31]. Recent findings suggest that the GluN2A subunit of NMDA receptors mediates these antidepressant effects [36], whereas loss-of-function mutations in this subunit significantly increase susceptibility to schizophrenia [39, 40].

Additionally, optogenetic and chemogenetic modulation of mPFC GABAergic interneuron activity has demonstrated the potential to mitigate behavioral abnormalities in models of depression and schizophrenia [14–16, 68]. However, these methods may not be suitable for therapeutic applications. In contrast, pharmacological inhibition of GluN2A could provide valuable insights into the microcircuit mechanisms underlying its role in schizophrenia and depression, potentially leading to novel therapeutic approaches.

Our findings reveal that GluN2A inhibition leads to the disinhibition of PYNs in the dorsomedial prefrontal cortex (dmPFC), which aligns with previous discoveries conducted using nonspecific NMDA receptor blockers [6, 22, 29, 57, 69, 70] and highlights the significant contribution of GluN2A-containing NMDA receptors in regulating neuronal activity in the dmPFC. We further demonstrated that this disinhibition is mediated by the hyperpolarization of PV-INs, indicating the tonic activation of GluN2A-containing NMDA receptors in these cells. Additionally, compared with SST-INs and PYNs, PV-INs receive more functional presynaptic excitatory input and are more responsive to presynaptic excitatory input. This glutamate innervation in PV-INs leads to greater depolarization, which in turn removes the magnesium block from GluN2A-containing NMDA receptors. Consequently, PV-INs emerge as the key locus for GluN2A antagonism, given their role in modulating the excitatory–inhibitory balance in the dmPFC.

### Enhanced efficacy of MPX-004 toward GluN2A-containing NMDA receptors

NVP is a compound that was initially thought to be a selective blocker of the GluN2A subunit [71]. However, its selectivity for GluN2A over GluN2B is suboptimal, with *K*_i_ values of 30 nM and 320 nM, respectively [45]. TCN-201 and MPX-004 are therefore currently the only GluN2A-selective NMDA receptor antagonists commercially available [54]. Our study, in conjunction with previous findings [46], demonstrated that MPX-004, a derivative of TCN-201, outperforms TCN-201 in terms of its efficacy in inhibiting GluN2A-containing NMDA receptors. While the effectiveness of TCN-201 is highly sensitive to glycine concentrations, limiting its practical application, MPX-004 maintains robust inhibition across a broader range of glycine concentrations. This improvement in pharmacological properties makes MPX-004 a more reliable tool for investigating the role of native GluN2A-containing NMDA receptors in neurological disorders.

### Antidepressant-like effects and deficits in sensorimotor gating mediated by MPX-004 treatment

We investigated behavioral outcomes following the local administration of MPX-004 to the dmPFC. While no changes were observed in the open field test, a significant reduction in immobility time was noted in the forced swim test, a widely recognized model for assessing depression. These findings suggest a promising antidepressant-like effect of MPX-004. Further evaluation of its antidepressant properties in mice using depression models, such as chronic unpredictable mild stress or chronic social defeat stress, would provide valuable insights into the potency and duration of the antidepressant effects of MPX-004.

In comparison, ketamine, a non-subtype-selective NMDA receptor blocker, produces rapid and sustained antidepressant effects at low doses in patients with depression [31]. However, significant links have been reported between the antidepressant properties of ketamine and its psychotomimetic effects in humans [72]. This relationship has also been replicated in rodent studies. For example, recent research demonstrated that the local administration of ketamine to the mPFC elicited antidepressant-like effects in both the forced swim test and the tail suspension test while also inducing psychotomimetic-like effects characterized by deficits in sensorimotor gating [73]. This latter effect is widely recognized as a crucial parameter for assessing schizophrenia [74].

MPX-004 induced deficits in sensorimotor gating but did not produce a hyperlocomotion phenotype. Interestingly, the local application of ketamine in the mPFC also did not result in hyperlocomotion [73], suggesting that the hyperlocomotion induced by ketamine administered intraperitoneally is not mediated by the prefrontal cortex.

Our finding that the pharmacological inhibition of prefrontal GluN2A-containing NMDA receptors may produce both antidepressant-like effects and schizophrenia-like behaviors aligns with clinical observations of ketamine, which can induce psychotomimetic side effects when used to treat major depressive disorder. The disinhibition of prefrontal pyramidal neurons may underlie both phenotypes, suggesting that the prefrontal cortex may not be the ideal target for therapeutic modulation.

### Neural circuit alterations and mechanisms

Ketamine has been shown to activate glutamatergic neurons by GABAergic disinhibition in the mPFC [73]. Our in vivo recordings and c-Fos staining experiments demonstrated that MPX-004 administration led to increased activity in PYNs and decreased activity in PV interneurons. This shift in the E/I balance was accompanied by antidepressant-like and schizophrenia-like behaviors. The elevated firing frequency of PYNs, alongside decreased PV-IN activity, suggests a disinhibition effect, which may contribute to the observed behavioral changes. These findings highlight the crucial role of GluN2A-containing NMDA receptors in modulating neural network activity in the dmPFC.

PV-INs are particularly sensitive to GluN2A antagonism, likely because of several factors. Consistent with previous findings that the ambient synaptic glutamate concentration is greater in interneurons than in pyramidal neurons [75], we observed tonic activation of GluN2A-containing NMDA receptors in PV-INs. Additionally, PV-INs receive excitatory postsynaptic potentials at higher frequencies, suggesting intense excitatory innervation of these interneurons. This intense excitatory input may make PV-INs more responsive to presynaptic input. Consequently, the more active state of PV-INs could lead to a depolarized, more excitable state, rendering them susceptible to GluN2A receptor inhibition. In support of this hypothesis, our in vivo recordings revealed that interneurons with relatively high firing frequencies presented reduced activity following MPX-004 infusion, whereas pyramidal neurons with lower firing rates presented increased activity after MPX-004 treatment. Together, these data demonstrate the mechanism underlying the disinhibition of the prefrontal cortex by MPX-004.

The modulation of PV-IN activity has been used to explore its effects on brain circuits and behavior. The ablation of NMDA receptors from PV-INs in mice increased cortical activity [26, 28, 70] and induced schizophrenia-like behaviors [26, 27]. The optogenetic inhibition or activation of as few as 10% of PV-INs in the barrel cortex led to corresponding increases or decreases in spontaneous neocortical activity [68]. The chemogenetic inhibition of PV-INs induced an increased firing frequency of PYNs in the cortex, suggesting strong inhibitory control of PV-INs over PYNs in vivo [11]. Additionally, the optogenetic inhibition or chemo-inhibition of PV-INs in the prefrontal cortex mitigated depressive behavior in a depression model [14, 16]. These findings further substantiate the critical role of PV-INs in shaping the E/I balance.

### Species considerations

In the present study, we evaluated the inhibitory effects of MPX-004 on rat NMDA receptors. However, subsequent experiments were conducted in mice, as the availability of genetic tools in this species allows for more detailed investigations of prefrontal cortical microcircuits. Notably, GluN1 differs by only two amino acids between rats and mice, whereas GluN2A differs at 15 positions. Importantly, the amino acid residues forming the MPX-004-binding pocket are fully conserved in rat and mouse GluN1/GluN2A receptors (Supplementary Fig. 4). This conservation suggests that the inhibitory effects of MPX-004 on GluN2A-containing NMDA receptors are likely preserved across these species. Nonetheless, we acknowledge that species-specific differences in nonconserved residues outside the binding site could influence receptor conformation or regulation, and we highlight this as a limitation and a potential direction for future research.

## CONCLUSION

In this study, we demonstrate that the GluN2A-selective NMDA receptor blocker MPX-004 induces acute behavioral effects similar to those of ketamine, producing both antidepressant-like and schizophrenia-like effects. Moreover, our findings provide deeper insights into the impact of acute inhibition of GluN2A-containing NMDA receptors on prefrontal cortex neurons and circuits. This enhances our understanding of the underlying neural mechanisms driving these behavioral outcomes.

## Supplementary Material

Supplemental Figure S1

Supplemental Figure S2

Supplemental Figure S3

Supplemental Figure S4

Supplemental Information

**Supplementary information** The online version contains supplementary material available at https://doi.org/10.1038/s41401-025-01643-2.

## Data Availability

The original data are available from FY upon request.

## References

[1] Pizzagalli DA, Roberts AC. Prefrontal cortex and depression. Neuropsychopharmacology. 2022;47:225–46. 34341498 10.1038/s41386-021-01101-7 PMC8617037

[2] Abdallah CG, Averill CL, Salas R, Averill LA, Baldwin PR, Krystal JH, Prefrontal connectivity and glutamate transmission: relevance to depression pathophysiology and ketamine treatment. Biol Psychiatry Cogn Neurosci Neuroimaging. 2017;2:566–74. 29034354 10.1016/j.bpsc.2017.04.006 PMC5635826

[3] Hare BD, Duman RS. Prefrontal cortex circuits in depression and anxiety: contribution of discrete neuronal populations and target regions. Mol Psychiatry. 2020;25:2742–58. 32086434 10.1038/s41380-020-0685-9 PMC7442605

[4] Gao WJ, Yang SS, Mack NR, Chamberlin LA. Aberrant maturation and connectivity of prefrontal cortex in schizophrenia-contribution of NMDA receptor development and hypofunction. Mol Psychiatry. 2022;27:731–43. 34163013 10.1038/s41380-021-01196-w PMC8695640

[5] Adell A Brain NMDA receptors in schizophrenia and depression. Biomolecules. 2020;10:947. 32585886 10.3390/biom10060947 PMC7355879

[6] Coyle JT, Ruzicka WB, Balu DT. Fifty years of research on schizophrenia: the ascendance of the glutamatergic synapse. Am J Psychiatry. 2020;177:1119–28. 33256439 10.1176/appi.ajp.2020.20101481 PMC8011846

[7] Coyle JT. NMDA receptor and schizophrenia: a brief history. Schizophr Bull. 2012;38:920–6. 22987850 10.1093/schbul/sbs076 PMC3446237

[8] Callicott JH, Bertolino A, Mattay VS, Langheim FJ, Duyn J, Coppola R, Physiological dysfunction of the dorsolateral prefrontal cortex in schizophrenia revisited. Cereb Cortex. 2000;10:1078–92. 11053229 10.1093/cercor/10.11.1078

[9] Foss-Feig JH, Adkinson BD, Ji JL, Yang G, Srihari VH, McPartland JC, Searching for cross-diagnostic convergence: neural mechanisms governing excitation and inhibition balance in schizophrenia and autism spectrum disorders. Biol Psychiatry. 2017;81:848–61. 28434615 10.1016/j.biopsych.2017.03.005 PMC5436134

[10] Ferguson BR, Gao WJ. PV interneurons: critical regulators of E/I balance for prefrontal cortex-dependent behavior and psychiatric disorders. Front Neural Circuits. 2018;12:37. 29867371 10.3389/fncir.2018.00037 PMC5964203

[11] Zhang CL, Koukouli F, Allegra M, Ortiz C, Kao HL, Maskos U, Inhibitory control of synaptic signals preceding locomotion in mouse frontal cortex. Cell Rep. 2021;37:110035. 34818555 10.1016/j.celrep.2021.110035 PMC8640223

[12] Etkin A, Egner T, Kalisch R. Emotional processing in anterior cingulate and medial prefrontal cortex. Trends Cogn Sci. 2011;15:85–93. 21167765 10.1016/j.tics.2010.11.004 PMC3035157

[13] Kumar S, Black SJ, Hultman R, Szabo ST, DeMaio KD, Du J, Cortical control of affective networks. J Neurosci. 2013;33:1116–29. 23325249 10.1523/JNEUROSCI.0092-12.2013 PMC3711588

[14] Fogaca MV, Wu M, Li C, Li XY, Picciotto MR, Duman RS. Inhibition of GABA interneurons in the mPFC is sufficient and necessary for rapid antidepressant responses. Mol Psychiatry. 2021;26:3277–91. 33070149 10.1038/s41380-020-00916-y PMC8052382

[15] Chamberlin LA, Yang S-S, McEachern EP, Lucas JT, McLeod Ii OW, Rolland CA, Pharmacogenetic activation of parvalbumin interneurons in the prefrontal cortex rescues cognitive deficits induced by adolescent MK801 administration. Neuropsychopharmacology. 2023;48:1267–76. 37041206 10.1038/s41386-023-01576-6 PMC10353985

[16] Nawreen N, Oshima K, Chambers J, Smail M, Herman JP. Inhibition of prefrontal cortex parvalbumin interneurons mitigates behavioral and physiological sequelae of chronic stress in male mice. Stress. 2024;27:2361238. 38962839 10.1080/10253890.2024.2361238 PMC11725266

[17] Dienel SJ, Lewis DA. Alterations in cortical interneurons and cognitive function in schizophrenia. Neurobiol Dis. 2019;131:104208. 29936230 10.1016/j.nbd.2018.06.020 PMC6309598

[18] Wang AY, Lohmann KM, Yang CK, Zimmerman EI, Pantazopoulos H, Herring N, Bipolar disorder type 1 and schizophrenia are accompanied by decreased density of parvalbumin- and somatostatin-positive interneurons in the parahippocampal region. Acta Neuropathol. 2011;122:615–26. 21968533 10.1007/s00401-011-0881-4 PMC4207060

[19] Traynelis SF, Wollmuth LP, McBain CJ, Menniti FS, Vance KM, Ogden KK, Glutamate receptor ion channels: structure, regulation, and function. Pharmacol Rev. 2010;62:405–96. 20716669 10.1124/pr.109.002451 PMC2964903

[20] Paoletti P, Bellone C, Zhou Q. NMDA receptor subunit diversity: impact on receptor properties, synaptic plasticity and disease. Nat Rev Neurosci. 2013;14:383–400. 23686171 10.1038/nrn3504

[21] Wang M, Yang Y, Wang CJ, Gamo NJ, Jin LE, Mazer JA, NMDA receptors subserve persistent neuronal firing during working memory in dorsolateral prefrontal cortex. Neuron. 2013;77:736–49. 23439125 10.1016/j.neuron.2012.12.032 PMC3584418

[22] Homayoun H, Moghaddam B. NMDA receptor hypofunction produces opposite effects on prefrontal cortex interneurons and pyramidal neurons. J Neurosci. 2007;27:11496–500. 17959792 10.1523/JNEUROSCI.2213-07.2007 PMC2954603

[23] Hansen KB, Wollmuth LP, Bowie D, Furukawa H, Menniti FS, Sobolevsky AI, Structure, function, and pharmacology of glutamate receptor ion channels. Pharmacol Rev. 2021;73:298–487. 34753794 10.1124/pharmrev.120.000131 PMC8626789

[24] Collingridge GL, Monaghan DT. The continually evolving role of NMDA receptors in neurobiology and disease. Neuropharmacology. 2022;210:109042. 35307365 10.1016/j.neuropharm.2022.109042

[25] Guo F, Zhang B, Shen F, Li Q, Song Y, Li T, Sevoflurane acts as an antidepressant by suppression of GluN2D-containing NMDA receptors on interneurons. Br J Pharmacol. 2024;181:3483–502. 38779864 10.1111/bph.16420

[26] Gawande DY, S Narasimhan KK, Shelkar GP, Pavuluri R, Stessman HAF, Dravid SM. GluN2D Subunit in parvalbumin interneurons regulates prefrontal cortex feed-forward inhibitory circuit and molecular networks relevant to schizophrenia. Biol Psychiatry. 2023;94:297–309. 37004850 10.1016/j.biopsych.2023.03.020 PMC10524289

[27] Belforte JE, Zsiros V, Sklar ER, Jiang Z, Yu G, Li Y, Postnatal NMDA receptor ablation in corticolimbic interneurons confers schizophrenia-like phenotypes. Nat Neurosci. 2010;13:76–83. 19915563 10.1038/nn.2447 PMC2797836

[28] Alvarez RJ, Pafundo DE, Zold CL, Belforte JE. Interneuron NMDA Receptor ablation induces hippocampus-prefrontal cortex functional hypoconnectivity after adolescence in a mouse model of schizophrenia. J Neurosci. 2020;40:3304–17. 32205341 10.1523/JNEUROSCI.1897-19.2020 PMC7159887

[29] Pittenger C, Sanacora G, Krystal JH. The NMDA receptor as a therapeutic target in major depressive disorder. CNS Neurol Disord Drug Targets. 2007;6:101–15. 17430148 10.2174/187152707780363267

[30] Olney JW, Newcomer JW, Farber NB. NMDA receptor hypofunction model of schizophrenia. J Psychiatr Res. 1999;33:523–33. 10628529 10.1016/s0022-3956(99)00029-1

[31] Berman RM, Cappiello A, Anand A, Oren DA, Heninger GR, Charney DS, Antidepressant effects of ketamine in depressed patients. Biol Psychiatry. 2000;47:351. 10686270 10.1016/s0006-3223(99)00230-9

[32] Gerhard DM, Pothula S, Liu RJ, Wu M, Li XY, Girgenti MJ, GABA interneurons are the cellular trigger for ketamine’s rapid antidepressant actions. J Clin Invest. 2020;130:1336–49. 31743111 10.1172/JCI130808 PMC7269589

[33] Ali F, Gerhard DM, Sweasy K, Pothula S, Pittenger C, Duman RS, Ketamine disinhibits dendrites and enhances calcium signals in prefrontal dendritic spines. Nat Commun. 2020;11:72. 31911591 10.1038/s41467-019-13809-8 PMC6946708

[34] Zanos P, Moaddel R, Morris PJ, Georgiou P, Fischell J, Elmer GI, NMDAR inhibition-independent antidepressant actions of ketamine metabolites. Nature. 2016;533:481–6. 27144355 10.1038/nature17998 PMC4922311

[35] Klein ME, Chandra J, Sheriff S, Malinow R. Opioid system is necessary but not sufficient for antidepressive actions of ketamine in rodents. Proc Natl Acad Sci USA. 2020;117:2656–62. 31941713 10.1073/pnas.1916570117 PMC7007545

[36] Su T, Lu Y, Fu C, Geng Y, Chen Y. GluN2A mediates ketamine-induced rapid antidepressant-like responses. Nat Neurosci. 2023;26:1751–61. 37709995 10.1038/s41593-023-01436-y

[37] Zanos P, Brown KA, Georgiou P, Yuan P, Zarate CA Jr, Thompson SM, NMDA Receptor activation-dependent antidepressant-relevant behavioral and synaptic actions of ketamine. J Neurosci. 2023;43:1038–50. 36596696 10.1523/JNEUROSCI.1316-22.2022 PMC9908316

[38] Wang G, Qi W, Liu QH, Guan W. GluN2A: A promising target for developing novel antidepressants. Int J Neuropsychopharmacol. 2024;27:pyae037. 39185814 10.1093/ijnp/pyae037 PMC12042802

[39] Singh T, Poterba T, Curtis D, Akil H, Al Eissa M, Barchas JD, Rare coding variants in ten genes confer substantial risk for schizophrenia. Nature. 2022;604:509–16. 35396579 10.1038/s41586-022-04556-w PMC9805802

[40] Trubetskoy V, Pardiñas AF, Qi T, Panagiotaropoulou G, Awasthi S, Bigdeli TB, Mapping genomic loci implicates genes and synaptic biology in schizophrenia. Nature. 2022;604:502–8. 35396580 10.1038/s41586-022-04434-5 PMC9392466

[41] Farsi Z, Nicolella A, Simmons SK, Aryal S, Shepard N, Brenner K, Brain-region-specific changes in neurons and glia and dysregulation of dopamine signaling in Grin2a mutant mice. Neuron. 2023;111:3378–96.e9. 37657442 10.1016/j.neuron.2023.08.004

[42] Herzog LE, Wang L, Yu E, Choi S, Farsi Z, Song BJ, Mouse mutants in schizophrenia risk genes GRIN2A and AKAP11 show EEG abnormalities in common with schizophrenia patients. Transl Psychiatry. 2023;13:92. 36914641 10.1038/s41398-023-02393-7 PMC10011509

[43] Lu Y, Mu L, Elstrott J, Fu C, Sun C, Su T, Differential depletion of GluN2A induces heterogeneous schizophrenia-related phenotypes in mice. Ebiomedicine. 2024;102:105045. 38471394 10.1016/j.ebiom.2024.105045 PMC10943646

[44] Anticevic A, Hu X, Xiao Y, Hu J, Li F, Bi F, Early-course unmedicated schizophrenia patients exhibit elevated prefrontal connectivity associated with longitudinal change. J Neurosci. 2015;35:267–86. 25568120 10.1523/JNEUROSCI.2310-14.2015 PMC4287147

[45] Lind GE, Mou TC, Tamborini L, Pomper MG, De Micheli C, Conti P, Structural basis of subunit selectivity for competitive NMDA receptor antagonists with preference for GluN2A over GluN2B subunits. Proc Natl Acad Sci USA. 2017;114:E6942–E51. 28760974 10.1073/pnas.1707752114 PMC5565460

[46] Yi F, Mou TC, Dorsett KN, Volkmann RA, Menniti FS, Sprang SR, Structural basis for negative allosteric modulation of GluN2A-Containing NMDA receptors. Neuron. 2016;91:1316–29. 27618671 10.1016/j.neuron.2016.08.014 PMC5033714

[47] Yi F, Bhattacharya S, Thompson CM, Traynelis SF, Hansen KB. Functional and pharmacological properties of triheteromeric GluN1/2B/2D NMDA receptors. J Physiol. 2019;597:5495–514. 31541561 10.1113/JP278168 PMC6858497

[48] Samara Z, Evers EA, Peeters F, Uylings HB, Rajkowska G, Ramaekers JG, Orbital and medial prefrontal cortex functional connectivity of major depression vulnerability and disease. Biol Psychiatry Cogn Neurosci Neuroimaging. 2018;3:348–57. 29628067 10.1016/j.bpsc.2018.01.004 PMC5894884

[49] Hansen KB, Ogden KK, Yuan H, Traynelis SF. Distinct functional and pharmacological properties of triheteromeric GluN1/GluN2A/GluN2B NMDA receptors. Neuron. 2014;81:1084–96. 24607230 10.1016/j.neuron.2014.01.035 PMC3957490

[50] Yi F, Zachariassen LG, Dorsett KN, Hansen KB. Properties of triheteromeric *N*-methyl-*D*-aspartate receptors containing two distinct GluN1 isoforms. Mol Pharmacol. 2018;93:453–67. 29483146 10.1124/mol.117.111427 PMC5878673

[51] Royer S, Zemelman BV, Losonczy A, Kim J, Chance F, Magee JC, Control of timing, rate and bursts of hippocampal place cells by dendritic and somatic inhibition. Nat Neurosci. 2012;15:769–75. 22446878 10.1038/nn.3077 PMC4919905

[52] Petersen PC, Siegle JH, Steinmetz NA, Mahallati S, Buzsáki G. CellExplorer: A framework for visualizing and characterizing single neurons. Neuron. 2021;109:3594–608.e2. 34592168 10.1016/j.neuron.2021.09.002 PMC8602784

[53] Chung JE, Magland JF, Barnett AH, Tolosa VM, Tooker AC, Lee KY, A fully automated approach to spike sorting. Neuron. 2017;95:1381–94.e6. 28910621 10.1016/j.neuron.2017.08.030 PMC5743236

[54] Hansen KB, Ogden KK, Traynelis SF. Subunit-selective allosteric inhibition of glycine binding to NMDA receptors. J Neurosci. 2012;32:6197–208. 22553026 10.1523/JNEUROSCI.5757-11.2012 PMC3355950

[55] Kim S-Y, Kaufman MJ, Cohen BM, Jensen JE, Coyle JT, Du F, In vivo brain glycine and glutamate concentrations in patients with first-episode psychosis measured by echo time–averaged proton magnetic resonance spectroscopy at 4T. Biol Psychiatry. 2018;83:484–91. 29031411 10.1016/j.biopsych.2017.08.022 PMC5809251

[56] Horio M, Kohno M, Fujita Y, Ishima T, Inoue R, Mori H, Levels of D-serine in the brain and peripheral organs of serine racemase (Srr) knock-out mice. Neurochem Int. 2011;59:853–9. 21906644 10.1016/j.neuint.2011.08.017

[57] Moghaddam B, Adams B, Verma A, Daly D. Activation of glutamatergic neurotransmission by ketamine: a novel step in the pathway from NMDA receptor blockade to dopaminergic and cognitive disruptions associated with the prefrontal cortex. J Neurosci. 1997;17:2921–7. 9092613 10.1523/JNEUROSCI.17-08-02921.1997 PMC6573099

[58] Harrison PJ, Bannerman DM. GRIN2A (NR2A): a gene contributing to glutamatergic involvement in schizophrenia. Mol Psychiatry. 2023;28:3568–72. 37736757 10.1038/s41380-023-02265-y PMC10730418

[59] Krystal JH, Karper LP, Seibyl JP, Freeman GK, Delaney R, Bremner JD, Sub-anesthetic effects of the noncompetitive NMDA antagonist, ketamine, in humans: psychotomimetic, perceptual, cognitive, and neuroendocrine responses. Arch Gen Psychiatry. 1994;51:199–214. 8122957 10.1001/archpsyc.1994.03950030035004

[60] Kovács KJ. Measurement of immediate‐early gene activation‐c‐fos and beyond. J Neuroendocrinol. 2008;20:665–72. 18601687 10.1111/j.1365-2826.2008.01734.x

[61] Tremblay R, Lee S, Rudy B. GABAergic Interneurons in the neocortex: from cellular properties to circuits. Neuron. 2016;91:260–92. 27477017 10.1016/j.neuron.2016.06.033 PMC4980915

[62] Shepard R, Coutellier L. Changes in the prefrontal glutamatergic and parvalbumin systems of mice exposed to unpredictable chronic stress. Mol Neurobiol. 2018;55:2591–602. 28421533 10.1007/s12035-017-0528-0

[63] Xu P, Peng J, Yuan T, Chen Z, He H, Wu Z, High-throughput mapping of single-neuron projection and molecular features by retrograde barcoded labeling. eLife. 2024;13:e85419. 38390967 10.7554/eLife.85419 PMC10914349

[64] Courtin J, Chaudun F, Rozeske RR, Karalis N, Gonzalez-Campo C, Wurtz H, Prefrontal parvalbumin interneurons shape neuronal activity to drive fear expression. Nature. 2014;505:92–6. 24256726 10.1038/nature12755

[65] Chen H, He T, Li M, Wang C, Guo C, Wang W, Cell-type-specific synaptic modulation of mAChR on SST and PV interneurons. Front Psychiatry. 2022;13:1070478. 36713928 10.3389/fpsyt.2022.1070478 PMC9877455

[66] Liu L, Xu H, Wang J, Li J, Tian Y, Zheng J, Cell type-differential modulation of prefrontal cortical GABAergic interneurons on low gamma rhythm and social interaction. Sci Adv. 2020;6:eaay4073. 32832654 10.1126/sciadv.aay4073 PMC7439507

[67] Xu P, Chen A, Li Y, Xing X, Lu H. Medial prefrontal cortex in neurological diseases. Physiol Genomics. 2019;51:432–42. 31373533 10.1152/physiolgenomics.00006.2019 PMC6766703

[68] Yang J-W, Prouvot P-H, Reyes-Puerta V, Stüttgen MC, Stroh A, Luhmann HJ. Optogenetic modulation of a minor fraction of parvalbumin-positive interneurons specifically affects spatiotemporal dynamics of spontaneous and sensory-evoked activity in mouse somatosensory cortex in vivo. Cereb Cortex. 2017;27:5784–803. 29040472 10.1093/cercor/bhx261 PMC5939210

[69] Heckers S, Konradi C. GABAergic mechanisms of hippocampal hyperactivity in schizophrenia. Schizophrenia Res. 2015;167:4–11. 10.1016/j.schres.2014.09.041 PMC4402105 25449711

[70] Billingslea EN, Tatard-Leitman VM, Anguiano J, Jutzeler CR, Suh J, Saunders JA, Parvalbumin cell ablation of NMDA-R1 causes increased resting network excitability with associated social and self-care deficits. Neuropsychopharmacology. 2014;39:1603–13. 24525709 10.1038/npp.2014.7 PMC4023157

[71] Auberson YP, Allgeier H, Bischoff S, Lingenhoehl K, Moretti R, Schmutz M. 5-Phosphonomethylquinoxalinediones as competitive NMDA receptor antagonists with a preference for the human 1A/2A, rather than 1A/2B receptor composition. Bioorg Med Chem Lett. 2002;12:1099–102. 11909726 10.1016/s0960-894x(02)00074-4

[72] Sos P, Klirova M, Novak T, Kohutova B, Horacek J, Palenicek T. Relationship of ketamine’s antidepressant and psychotomimetic effects in unipolar depression. Neuro Endocrinol Lett. 2013;34:287–93. 23803871

[73] Zhang B, Yang X, Ye L, Liu R, Ye B, Du W, Ketamine activated glutamatergic neurotransmission by GABAergic disinhibition in the medial prefrontal cortex. Neuropharmacology. 2021;194:108382. 33144117 10.1016/j.neuropharm.2020.108382

[74] San-Martin R, Castro LA, Menezes PR, Fraga FJ, Simoes PW, Salum C. Meta-analysis of sensorimotor gating deficits in patients with schizophrenia evaluated by prepulse inhibition test. Schizophr Bull. 2020;46:1482–97. 32506125 10.1093/schbul/sbaa059 PMC8061122

[75] Yao L, Grand T, Hanson J, Paoletti P, Zhou Q. Higher ambient synaptic glutamate at inhibitory versus excitatory neurons differentially impacts NMDA receptor activity. Nat Commun. 2018;9:4000. 30275542 10.1038/s41467-018-06512-7 PMC6167324

