# Correction: GluN2A-NMDA receptor inhibition disinhibits the prefrontal cortex, reduces forced swim immobility, and impairs sensorimotor gating

**DOI:** 10.1038/s41401-025-01697-2

**Published:** 2025-11-04

**Authors:** Yuan-ping Dong, Yun Wu, Yi-lu Zhao, Yu-min Chen, Tong-ye Liu, Yi-he Zhang, Jie-ying Xie, Jin-feng Zhang, Han Zhang, He Chen, Yu Peng, Chun-lei Zhang, Andrew R. Rau, Kasper B. Hansen, Hai-bing Xu, Feng Yi

**Affiliations:** 1 Key Laboratory of Mental Health of the Ministry of Education, Guangdong-Hong Kong-Macao Greater Bay Area Center for Brain Science and Brain-Inspired Intelligence, Guangdong-Hong Kong Joint Laboratory for Psychiatric Disorders, Guangdong Province Key Laboratory of Psychiatric Disorders, Guangdong Basic Research Center of Excellence for Integrated Traditional and Western Medicine for Qingzhi Diseases, Department of Neurobiology, School of Basic Medical Sciences, Southern Medical University, Guangzhou, 510515 China; 2 Department of Neurology, Guangzhou First People’s Hospital, School of Medicine, South China University of Technology, Guangzhou, 510006 China; 3 Institut Pasteur, Université de Paris, Neural Circuits for Spatial Navigation and Memory, F-75015 Paris, France; 4 Center for Structural and Functional Neuroscience, Center for Biomolecular Structure and Dynamics, Division of Biological Sciences, University of Montana, Missoula, MT USA

Correction to: *Acta Pharmacologica Sinica* 10.1038/s41401-025-01643-2, published online 10 September 2025

The article “GluN2A-NMDA receptor inhibition disinhibits the prefrontal cortex, reduces forced swim immobility, and impairs sensorimotor gating”, written by Yuan-ping Dong, Yun Wu, Yi-lu Zhao, Yu-min Chen, Tong-ye Liu et al., was originally published under exclusive license to “The Author(s) under exclusive licence to The Shanghai Institute of Materia Medica, Chinese Academy of Sciences and Chinese Pharmacological Society 2025”. As a result of the subsequent decision to publish the article under the open access model, the article’s copyright notice was changed on 07 October 2025 to “© The Author(s) 2025” and the article is now distributed under a Creative Commons Attribution 4.0 International License, which permits use, sharing, adaptation, distribution and reproduction in any medium or format, as long as you give appropriate credit to the original author(s) and the source, provide a link to the Creative Commons licence, and indicate if changes were made. The images or other third party material in this article are included in the article’s Creative Commons licence, unless indicated otherwise in a credit line to the material. If material is not included in the article’s Creative Commons licence and your intended use is not permitted by statutory regulation or exceeds the permitted use, you will need to obtain permission directly from the copyright holder. To view a copy of this licence, visit http://creativecommons.org/licenses/by/4.0.

The original article has been corrected.

