# Supplementary figures and images for: GluN2A-NMDA receptor inhibition disinhibits the prefrontal cortex, reduces forced swim immobility, and impairs sensorimotor gating
Source: Acta Pharmacol Sin. 2025 Sep 10;47(1):53–68. doi: 10.1038/s41401-025-01643-2 (PMC12633600; doi:10.1038/s41401-025-01643-2)

## Supplemental Figure 1

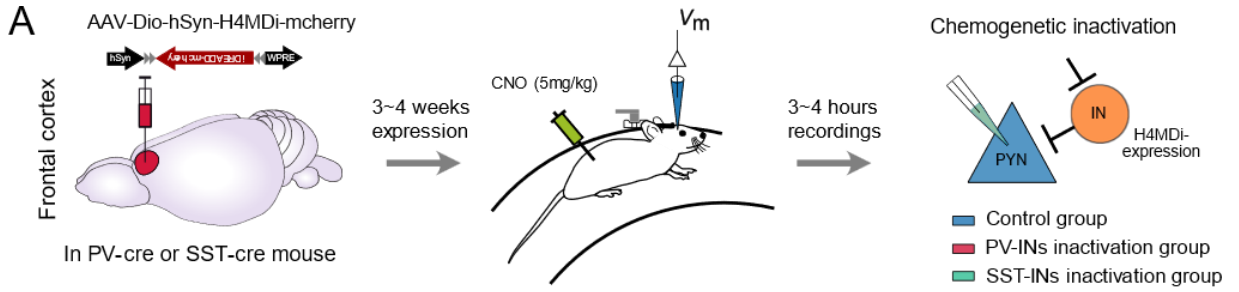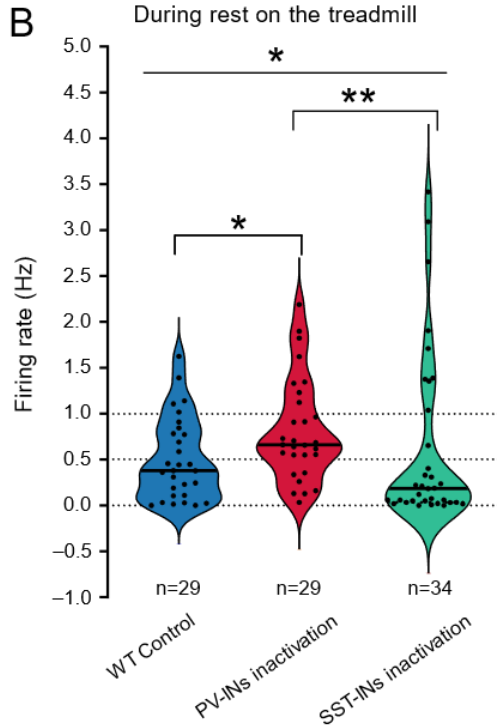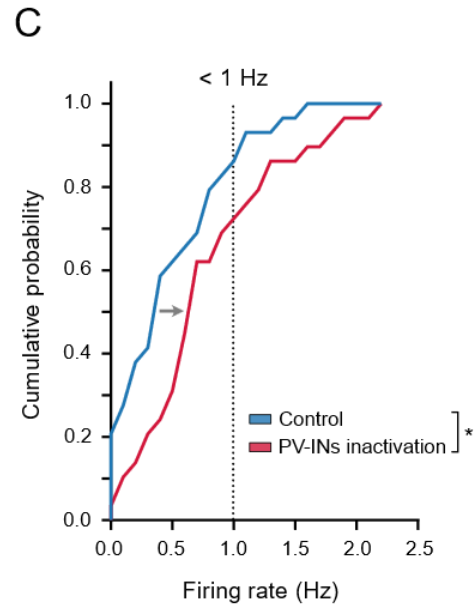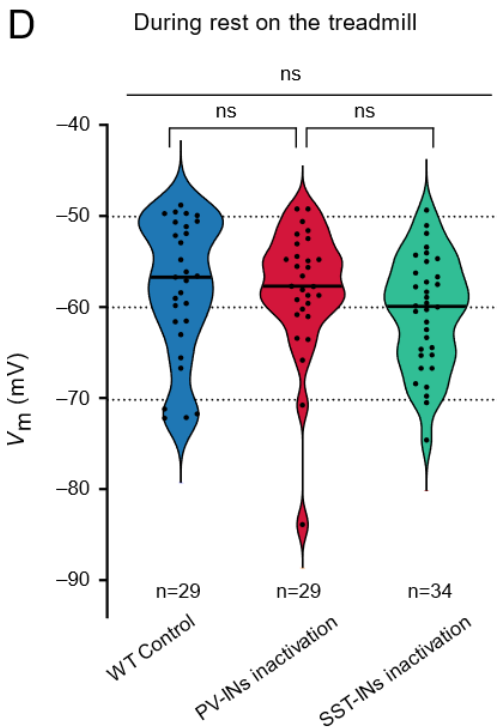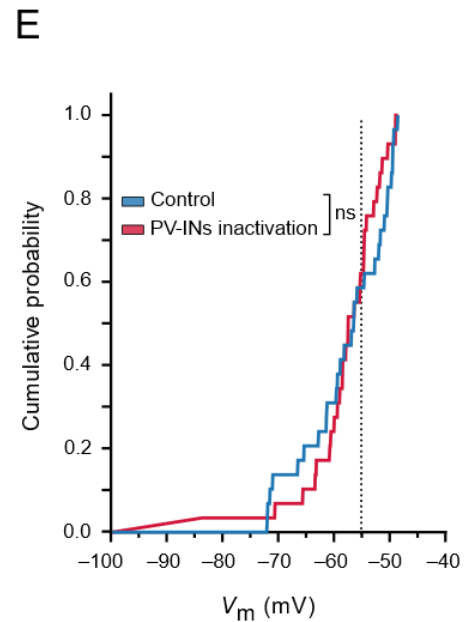

Supplement: Supplementary file 1 — Supplemental Figure 1 [file 41401_2025_1643_MOESM1_ESM.pdf]

Supplemental Figure 2

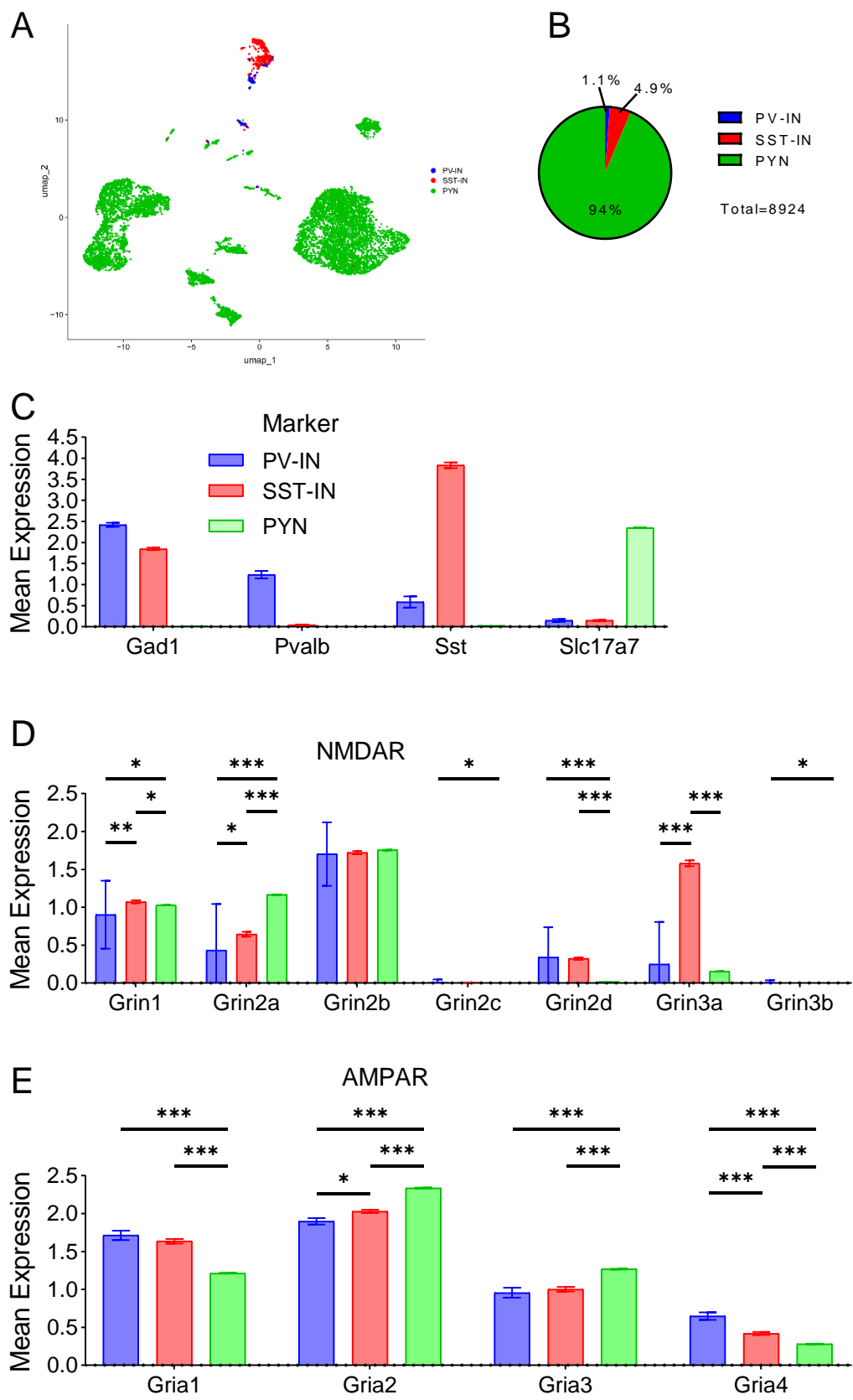

Supplement: Supplementary file 2 — Supplemental Figure 2 [file 41401_2025_1643_MOESM2_ESM.pdf]

Supplemental Figure 3

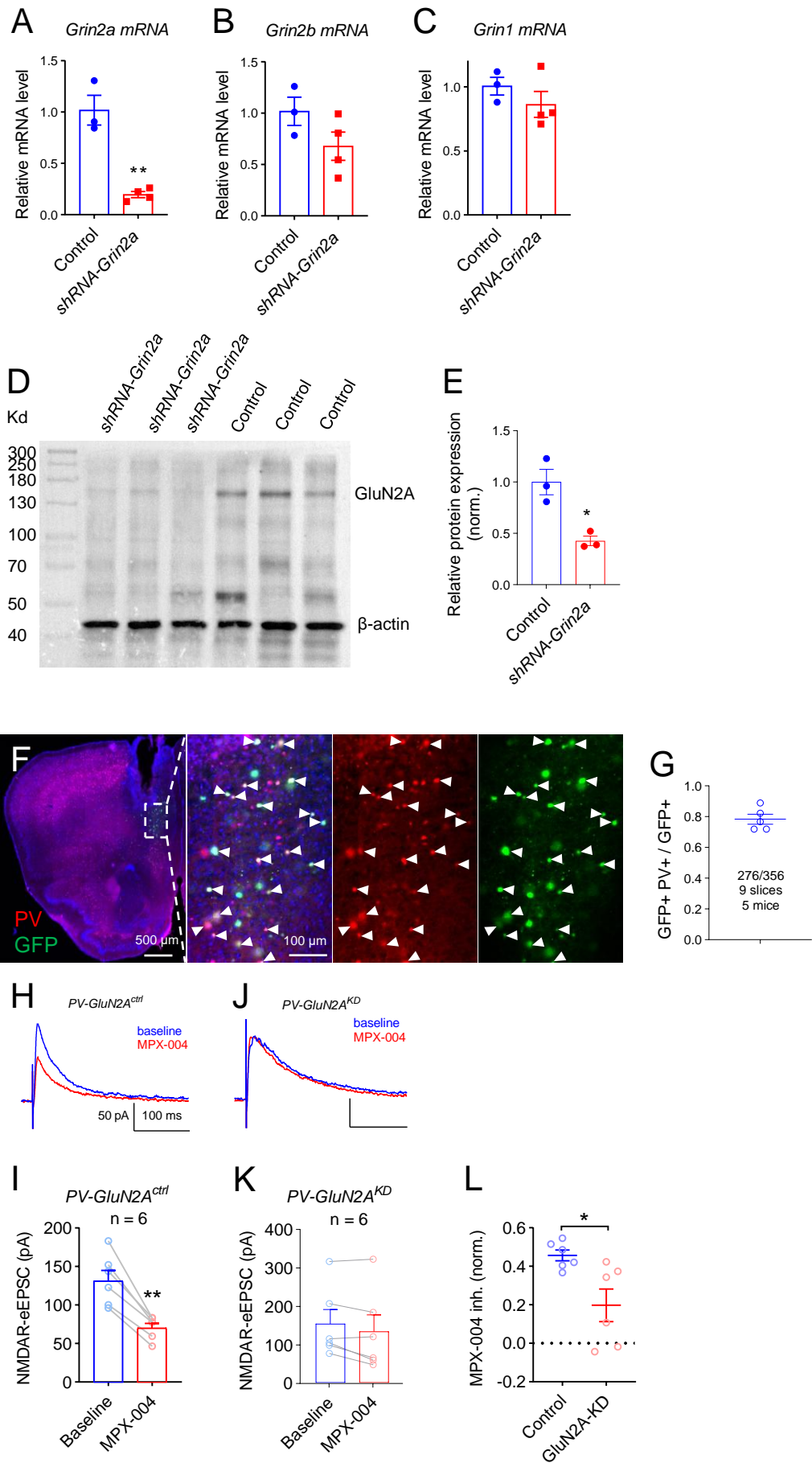

Supplement: Supplementary file 3 — Supplemental Figure 3 [file 41401_2025_1643_MOESM3_ESM.pdf]

Supplemental Figure 4

A

|            | Rat | Mouse |
|------------|-----|-------|
| GluN1-213  | E   | D     |
| GluN1-460  | I   | V     |
| GluN2A-324 | A   | T     |
| GluN2A-707 | R   | K     |

B

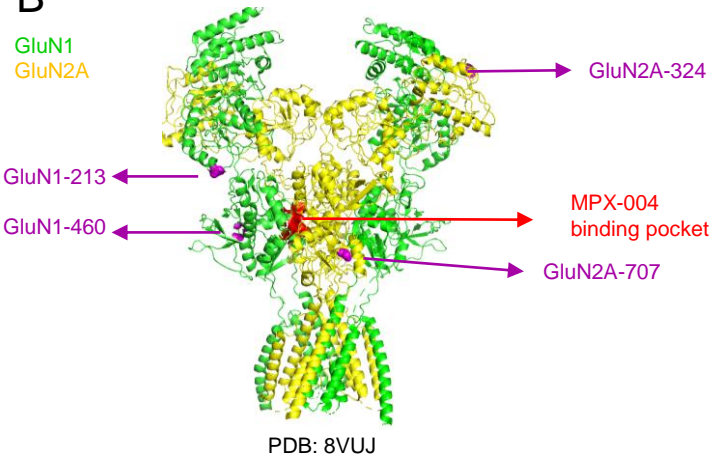

Supplement: Supplementary file 4 — Supplemental Figure 4 [file 41401_2025_1643_MOESM4_ESM.pdf]
