# Supplementary material for: GluN2A-NMDA receptor inhibition disinhibits the prefrontal cortex, reduces forced swim immobility, and impairs sensorimotor gating
Source: Acta Pharmacol Sin. 2025 Sep 10;47(1):53–68. doi: 10.1038/s41401-025-01643-2 (PMC12633600; doi:10.1038/s41401-025-01643-2)
Supplement: Supplementary file 5 — Supplemental Information [file 41401_2025_1643_MOESM5_ESM.docx]

**Supplemental Figure 1. Inactivation of PV-INs significantly enhances the firing rate of PYNs in the frontal cortex in awake mice (related to Figure 3).** (**A**) Schematic drawing of the experimental paradigm. PV-INs (red) or SOM-INs (green) were chemogenetically inactivated in the medial secondary motor cortex (MOs or M2), a subdivision of the rodent prefrontal cortex, using Cre-dependent expression of the inhibitory DREADD h4MDi in PV-Cre or SST-Cre mice. Clozapine (CNO; 5 mg/kg) was administered by intraperitoneal injection 30 min prior to *in vivo* electrophysiological recordings. The figures were partly adapted from images produced using scidraw.io, which are licensed under CC BY 4.0. (**B**) Comparison of firing rates under control conditions (n = 29 recordings; median value = 0.38 Hz; mean value = 0.50 Hz) and during chemogenetic inactivation of PV-INs (n = 29 recordings; median value = 0.66 Hz; mean value = 0.82 Hz) or SST-INs (n = 34 recordings; median value = 0.18 Hz; mean value = 0.63 Hz) during resting periods on the treadmill. Kruskal‒Wallis test, **p =* 0.0123. Control versus inactivation of PV-INs, Mann‒Whitney U test, **p =* 0.0241. Inactivation of PV-INs versus inactivation of SST-INs, Mann‒Whitney U test, ***p =* 0.0061. Control versus inactivation of SST-INs, n.s. Note that these results represent the same data as shown in Figure 5D (Zhang et al., 2021). (**C**) Cumulative distribution of the firing rate under control conditions and during inactivation of PV-INs. Kolmogorov–Smirnov test, **p =* 0.0308. (**D**) Comparison of the mean membrane potential under control conditions (median value = -56.7 mV; mean value = -58.0 mV) and during chemogenetic inactivation of PV-INs (median value = -57.7 mV; mean value = -58.1 mV) or SST-INs (median value = -60.0 mV; mean value = -60.1 mV) during the resting period on the treadmill. Kruskal‒Wallis test, n.s. Control versus inactivation of PV-INs; Mann‒Whitney U test, n.s. Inactivation of PV-INs versus inactivation of SST-INs; Mann‒Whitney U test, n.s. Note that these results represent the same data as shown in Figure 5E (Zhang et al., 2021). (**E**) Cumulative distribution of the membrane potential under control conditions and during the inactivation of PV-INs. Kolmogorov–Smirnov test, n.s.

**Supplemental Figure 2. Single-cell transcriptomic profiling of neurons in the PFC.** (**A**) UMAP graph showing the datasets containing PV-IN, SST-IN and PYN signals, which were collected from the vmPFC. PV-INs, *Pvalb*-positive interneurons; SST-INs, *Sst*-positive interneurons; PYNs, excitatory pyramidal neurons. (**B**) Pie graph showing the proportions of the three clusters of neurons involved in the analysis. (**C**) Bar graph showing the expression levels of marker genes (*Gad1,* *Pvalb*, *Sst,* and *Slc17a7*) used to confirm the three clusters. (**D**) Bar graph showing the expression of seven genes encoding NMDARs (*Grin1*, *Grin2a*, *Grin2b*, *Grin2c*, *Grin2d, Grin3a,* and *Grin3b*) and indicating greater expression of *Grin2a* in SST-INs than in PV-INs. (**E)** Bar graph showing the expression of four genes encoding AMPARs (*Gria1*, *Gria2*, *Gria3*, and *Gria4*). The data are presented as the means ± SEMs. Statistical significance was assessed using the Wilcoxon rank-sum test. Significant differences are indicated by asterisks (**p <* 0.05, ***p <* 0.01, ****p <* 0.001).

**Supplemental Figure 3. Verification of GluN2A knockdown efficiency.** (**A-C**) Bar graphs showing that infection with AAV-Dio-*Grin2a*-*shRNA-GFP* in the prefrontal cortex specifically and significantly reduced *Grin2a* mRNA levels (n = 3, 4 mice for each group, unpaired *t* test, ***p* = 0.0013) without affecting *Grin2b* or *Grin1* mRNA levels (*p* > 0.05). (**D-E**) Western blot analysis demonstrating reduced GluN2A protein expression following AAV-Grin2a-shRNA infection (n = 3 mice per group, unpaired *t* test, **p* = 0.0121). (**F**) Representative images showing GFP+ cells and PV-positive cells in the PFC. (**G**) Bar graph showing that most GFP+ cells were also stained with an anti-PV antibody. (**H**) Representative traces of NMDAR-mediated EPSCs in PV interneurons under baseline conditions and during MPX-004 application. (**I**) Bar graph showing that 10 µM MPX-004 significantly reduced the amplitude of NMDAR-EPSCs in PV interneurons. (**J**) Representative traces of NMDAR-EPSCs in PV interneurons with GluN2A knockdown (AAV-*Grin2a*-shRNA infection) under baseline conditions and following MPX-004 application (132.9 ± 13.6 pA vs. 71.4 ± 6.2 pA for baseline vs. MPX-004, n = 6; paired *t* test, ***p =* 0.0011). (**K**) Bar graph showing that MPX-004 treatment did not alter the NMDAR-EPSC amplitude in GluN2A-knockdown PV interneurons. (**L**) Bar graph showing that the proportion of NMDAR-EPSC inhibition by MPX-004 was significantly reduced in GluN2A-knockdown cells, confirming effective knockdown by AAV-*Grin2a*-shRNA (0.47 ± 0.03 vs. 0.20 ± 0.08 for control vs. GluN2A-KD, n = 6 per group; unpaired *t* test, **p =* 0.016).

**Supplemental Figure 4. Amino acids that differ between rat and mouse GluN1/2A are located far from the MPX-004-binding pocket.** (**A**) Two amino acids that differ between rat and mouse GluN1 are located in the extracellular domain (magenta). Among the fifteen amino acids that differ between rat and mouse GluN2A, only two are located in the extracellular domain (magenta). (**B**) The MPX-004-binding pocket (red) is situated at the dimer interface of the GluN1/2A agonist-binding domain, which is distant from the amino acids differing between rat and mouse GluN1/2A. The GluN1/2A structure was adapted from PDB 8VUJ, a human GluN1/2A structure.

Supplemental tables

Supplemental Table 1: Abbreviation list

| Abbreviation | Full name |
| --- | --- |
| ACSF | Artificial cerebrospinal fluid |
| dmPFC | Dorsomedial PFC |
| EPSC | Excitatory postsynaptic current |
| FST | Forced swim test |
| I-E-I | Inter-event interval |
| IPSC | Inhibitory postsynaptic current |
| NMDA | N-methyl D-aspartate acid |
| OFT | Open field test |
| PFC | Prefrontal cortex |
| PPI | Prepulse inhibition |
| PV-IN | Parvalbumin-expressing interneuron |
| PYN | Pyramidal neuron |
| sEPSC | Spontaneous excitatory postsynaptic current |
| sIPSC | Spontaneous inhibitory postsynaptic current |
| SST-IN | Somatostatin-expressing interneuron |

Supplemental Table 2: Primer list for qPCR.

| Primer | Primer sequence |
| --- | --- |
| GluN1-F-mouse-q | AGAATGTGACTCCCGCAGCAATG |
| GluN1-R-mouse-q | GGGCATCCTTGTGTCGCTTGTAG |
| GluN2A-F-mouse-q | ATATCGGCAGAACTCCACGCATTG |
| GluN2A-R-mouse-q | CATCCGCAGACAGGCATCACAC |
| GluN2B-F-mouse-q | GGCAAGCCTGGCATGGTCTTC |
| GluN2B-R-mouse-q | GGAGCAAGCGTAGGATATTGGAGTG |

Supplemental Table 3, linked to Figure 1

| Fig. 1 | measurement | GluN1/2A | | | GluN1/2A/2B | | | GluN1/2B | | |
| --- | --- | --- | --- | --- | --- | --- | --- | --- | --- | --- |
|  |  | mean | sem | n | mean | sem | n | mean | sem | n |
| Fig. 1c TCN | gly 1 µM | 99.4 | 0.1 | 5 | 87.3 | 2.0 | 5 | -2.0 | 3.1 | 5 |
|  | gly 3 µM | 97.6 | 0.5 | 5 | 77.9 | 2.0 | 5 | 0.0 | 2.5 | 5 |
|  | gly 10 µM | 84.7 | 1.3 | 5 | 54.5 | 4.0 | 5 | -4.7 | 1.4 | 5 |
|  | gly 30 µM | 59.6 | 3.0 | 5 | 37.4 | 4.3 | 6 | -1.4 | 3.2 | 5 |
| Fig. 1c MPX | gly 1 µM | 99.6 | 0.2 | 5 | 97.1 | 1.0 | 5 | 1.7 | 2.4 | 5 |
|  | gly 3 µM | 99.3 | 0.4 | 5 | 91.8 | 1.8 | 5 | 1.3 | 2.2 | 5 |
|  | gly 10 µM | 95.0 | 1.0 | 5 | 83.5 | 2.4 | 6 | -0.4 | 2.6 | 5 |
|  | gly 30 µM | 84.6 | 1.8 | 5 | 75.0 | 2.7 | 6 | -1.7 | 4.6 | 5 |
| Fig. 1d TCN | baseline | 0.068 | 0.005 | 5 | 0.083 | 0.003 | 6 | 0.607 | 0.016 | 5 |
|  | +TCN | 0.064 | 0.007 | 5 | 0.078 | 0.008 | 6 | 0.593 | 0.032 | 5 |
| Fig. 1c MPX | baseline | 0.072 | 0.004 | 5 | 0.068 | 0.004 | 6 | 0.556 | 0.052 | 5 |
|  | +MPX | 0.070 | 0.007 | 5 | 0.067 | 0.007 | 6 | 0.602 | 0.030 | 5 |

Supplemental Table 4, linked to Figure 2

| Fig. 2 | measurement | veh. | | | MPX | | | statistics | | | |
| --- | --- | --- | --- | --- | --- | --- | --- | --- | --- | --- | --- |
|  |  | mean | sem | n | mean | sem | n | normal distribution | test | Value | p |
| Fig. 2c | Duration in Center (s) | 43.58 | 3.56 | 13 | 44.72 | 5.43 | 14 | yes | unpaired t test | df(25)=0.01760 | 0.8644 |
| Fig. 2d | Total distance (m) | 24.65 | 1.50 | 13 | 23.00 | 1.72 | 14 | yes | unpaired t test | df(25)=0.6149 | 0.4788 |
| Fig. 2e | Distance 0-5 min (m) | 13.37 | 0.95 | 13 | 12.99 | 0.97 | 14 |  | multiple t test | df(25)=0.2776 | 0.7836 |
|  | Distance 5-10 min (m) | 11.28 | 0.86 | 13 | 10.01 | 0.82 | 14 |  |  | df(25)=1.07 | 0.2946 |
|  | Distance 10-15 min (m) | 9.02 | 0.82 | 13 | 8.27 | 0.71 | 14 |  |  | df(25)=0.6998 | 0.4905 |
|  | Distance 15-20 min (m) | 9.57 | 0.96 | 13 | 7.67 | 0.70 | 14 |  |  | df(25)=1.621 | 0.1177 |
|  | Distance 20-25 min (m) | 8.18 | 0.80 | 13 | 6.56 | 0.46 | 14 |  |  | df(25)=1.795 | 0.0847 |
|  | Distance 25-30 min (m) | 7.00 | 0.86 | 13 | 5.56 | 0.57 | 14 |  |  | df(25)=1.417 | 0.1689 |
| Fig. 2f | Total distance (m) | 58.43 | 4.08 | 13 | 51.04 | 3.60 | 14 | yes | unpaired t test | df(25)=1.362 | 0.1854 |
| Fig. 2g | resp.65 dB | 14.65 | 0.80 | 13 | 14.67 | 1.16 | 14 | yes | unpaired t test | df(25)=0.01760 | 0.9861 |
| Fig. 2h | resp.120 dB | 952.50 | 84.04 | 13 | 878.60 | 85.62 | 14 | yes | unpaired t test | df(25)=0.6149 | 0.5442 |
| Fig. 2i | 78 dB | 0.12 | 0.03 | 13 | 0.04 | 0.03 | 14 | - | Two-way ANOVA | F (1, 100) = 13.25, p=0.0004 for treatment, followed by Two-stage linear step-up procedure of Benjamini, Krieger and Yekutieli | 0.1265 |
|  | 82 dB | 0.24 | 0.04 | 13 | 0.16 | 0.03 | 14 | - |  |  | 0.1497 |
|  | 86 dB | 0.36 | 0.04 | 13 | 0.24 | 0.04 | 14 | - |  |  | 0.0330 |
|  | 90 dB | 0.39 | 0.04 | 13 | 0.27 | 0.05 | 14 | - |  |  | 0.0360 |
| Fig. 2j | Immobile (s) | 92.24 | 11.99 | 13 | 65.46 | 5.74 | 14 | yes | unpaired t test | df(25)=2.062 | 0.0497 |

Supplemental Table 5, linked to Figure 3

| Fig. 3 | measurement | veh. | | | MPX | | | statistics | | | |
| --- | --- | --- | --- | --- | --- | --- | --- | --- | --- | --- | --- |
|  |  | mean | sem | n | mean | sem | n | normal distribution | test | Value | p |
| Fig. 3b | Firing rate (Hz) | 2.72 | 0.22 | 130 | 1.76 | 0.15 | 130 | no | Wilcoxon matched-pairs signed-rank test | W=-4185 | <0.0001 |
| Fig. 3d | Firing rate (Hz) | 0.55 | 0.05 | 31 | 1.10 | 0.22 | 31 | no | Wilcoxon matched-pairs signed-rank test | W=242 | 0.0167 |
| Fig. 3e | Firing rate (Hz) | 3.39 | 0.25 | 99 | 1.96 | 0.18 | 99 | no | Wilcoxon matched-pairs signed-rank test | W=-3544 | <0.0001 |
| Fig. 3h | Firing rate (Hz) | 2.71 | 0.42 | 43 | 1.94 | 0.30 | 43 | no | Wilcoxon matched-pairs signed-rank test | W=-352 | 0.0331 |
| Fig. 3j | Firing rate (Hz) | 0.57 | 0.06 | 12 | 0.97 | 0.27 | 12 | no | Wilcoxon matched-pairs signed-rank test | W=30 | 0.2661 |
| Fig. 3k | Firing rate (Hz) | 3.54 | 0.51 | 31 | 2.32 | 0.39 | 31 | no | Wilcoxon matched-pairs signed-rank test | W=-298 | 0.0027 |
| Fig. 3l | Firing rate (Hz) | 2.72 | 0.26 | 87 | 1.66 | 0.17 | 87 | no | Wilcoxon matched-pairs signed-rank test | W=-2074 | <0.0001 |
| Fig. 3m | Firing rate (Hz) | 0.54 | 0.08 | 19 | 1.18 | 0.32 | 19 | no | Wilcoxon matched-pairs signed-rank test | W=104 | 0.0361 |
| Fig. 3o | Firing rate (Hz) | 3.33 | 0.29 | 68 | 1.80 | 0.20 | 68 | no | Wilcoxon matched-pairs signed-rank test | W=-1774 | <0.0001 |

Supplemental Table 6, linked to Figure 4

| Fig. 4 | measurement | veh. | | | MPX | | | statistics | | | |
| --- | --- | --- | --- | --- | --- | --- | --- | --- | --- | --- | --- |
|  |  | mean | sem | n | mean | sem | n | normal distribution | test | Value | p |
| Fig. 4j | PV | 0.0081 | 0.0019 | 11 | 0.0057 | 0.0008 | 13 | yes | Unpaired t test | t(22)=1.218 | 0.2363 |
| Fig. 4j | PV by mouse | 0.0080 | 0.0015 | 3 | 0.0057 | 0.0007 | 3 |  | Unpaired t test | t(4)=1.376 | 0.2434 |
| Fig. 4j | SST | 0.0216 | 0.0021 | 13 | 0.0178 | 0.0019 | 12 | yes | Unpaired t test | t(23)=1.333 | 0.1956 |
| Fig. 4j | SST by mouse | 0.0218 | 0.0018 | 3 | 0.0178 | 0.0029 | 3 |  | Unpaired t test | t(4)=1.190 | 0.2998 |
| Fig. 4j | CaMKII | 0.0132 | 0.0043 | 7 | 0.0188 | 0.0044 | 9 | yes | Unpaired t test | t(14)=0.8964 | 0.3852 |
| Fig. 4j | CaMKII by mouse | 0.0128 | 0.0042 | 3 | 0.0188 | 0.0026 | 3 |  | Unpaired t test | t(4)=1.215 | 0.2912 |
| Fig. 4k | PV | 0.0061 | 0.0012 | 12 | 0.0024 | 0.0005 | 13 | no | Mann‒Whitney U test | U=27 | 0.0045 |
| Fig. 4k | PV by mouse | 0.0060 | 0.0005 | 3 | 0.0025 | 0.0008 | 3 |  | Unpaired t test | t(4)=3.906 | 0.0175 |
| Fig. 4k | SST | 0.0171 | 0.0013 | 13 | 0.0163 | 0.0018 | 12 | no | Mann‒Whitney U test | U=68 | 0.6114 |
| Fig. 4k | SST by mouse | 0.0170 | 0.0009 | 3 | 0.0163 | 0.0026 | 3 |  | Unpaired t test | t(4)=0.2768 | 0.7956 |
| Fig. 4k | CaMKII | 0.0055 | 0.0011 | 7 | 0.0105 | 0.0017 | 9 | yes | Unpaired t test | t(14)=2.347 | 0.0342 |
| Fig. 4k | CaMKII by mouse | 0.0056 | 0.0004 | 3 | 0.0108 | 0.0010 | 3 |  | Unpaired t test | t(4)=4.699 | 0.0093 |
| Fig. 4n | sIPSC amp. | 13.65 | 1.32 | 8 | 13.49 | 0.78 | 10 | no | Mann‒Whitney U test | U=37 | 0.8286 |
| Fig. 4n | amp. by mouse | 13.62 | 0.88 | 3 | 13.53 | 0.91 | 3 |  | Unpaired t test | t(4)=0.07133 | 0.9466 |
| Fig. 4n | sIPSC freq. | 11.85 | 2.20 | 8 | 3.59 | 0.73 | 10 | yes | Unpaired t test | t(16)=3.895 | 0.0013 |
| Fig. 4n | freq. by mouse | 11.75 | 1.23 | 3 | 3.53 | 0.41 | 3 |  | Unpaired t test | t(4)=6.356 | 0.0031 |
| Fig. 4q | sEPSC amp. | -9.99 | 1.19 | 9 | -11.18 | 0.98 | 10 | yes | Unpaired t test | t(17)=0.779 | 0.4467 |
| Fig. 4q | amp. by mouse | -9.99 | 1.58 | 3 | -10.91 | 1.42 | 3 |  | Unpaired t test | t(4)=0.4321 | 0.6880 |
| Fig. 4q | sEPSC freq. | 3.47 | 0.8716 | 9 | 7.23 | 1.476 | 10 | yes | Unpaired t test | t(17)=2.131 | 0.0480 |
| Fig. 4q | freq. by mouse | 3.47 | 0.91 | 3 | 7.36 | 1.06 | 3 |  | Unpaired t test | t(4)=02.787 | 0.0494 |

Data with a gray background represent statistical data based on values averaged across individual mice.

Supplemental Table 7, linked to Figure 5

| Fig. 5 | measurement | veh. | | | MPX | | | statistics | | | |
| --- | --- | --- | --- | --- | --- | --- | --- | --- | --- | --- | --- |
|  |  | mean | sem | n | mean | sem | n | normal distribution | test | Value | p |
| Fig. 5c | Amp. (pA) | 131.3 | 17.4 | 7 | 93.1 | 11.8 | 7 | yes | Paired t test | t(6)=5.038 | 0.0024 |
| Fig. 5c | Amp. (pA) | 132 | 13.51 | 3 | 94.46 | 9.02 | 3 |  | Paired t test | t(2)=5.483 | 0.0230 |
| Fig. 5f | Amp. (pA) | 90.3 | 14.6 | 6 | 69.9 | 15.0 | 6 | no | Wilcoxon matched-pairs signed-rank test | W=-21 | 0.0313 |
| Fig. 5f | Amp. (pA) | 90.33 | 12.49 | 3 | 69.88 | 13.32 | 3 |  | Paired t test | t(2)=4.716 | 0.0421 |
| Fig. 5g | MPX inh. | 0.281 | 0.030 | 7 | 0.267 | 0.063 | 6 | yes | Unpaired t test | t(11)=0.1965 | 0.8478 |
| Fig. 5g | MPX inh. | 0.278 | 0.0122 | 3 | 0.267 | 0.06 | 3 |  | Unpaired t test | t(4)=0.1765 | 0.8685 |
| Fig. 5h | PV | 0 | 0 | 6 | -25.89 | 9.245 | 6 | - | Two-way ANOVA | F (1, 10) = 6.293, p=0.031 for treatment, followed by Sidak's multiple-comparisons test | 0.0397 |
|  | SST | 0 | 0 | 6 | -7.36 | 9.496 | 6 | - |  |  | 0.698 |
| Fig. 5i | PV | 0.091 | 0.0114 | 6 | 0.105 | 0.011 | 6 | - | Two-way ANOVA | F (1, 10) = 25.17, p=0.0005 for treatment, followed by Sidak's multiple-comparisons test | 0.034 |
|  | SST | 0.14 | 0.0235 | 6 | 0.16 | 0.025 | 6 | - |  |  | 0.003 |

Data with a gray background represent statistical data based on values averaged across individual mice.

Supplemental Table 8, linked to Figure 6

| Fig. 6 | measurement | PV | | | SST | | | PYN | | | statistics | | | |
| --- | --- | --- | --- | --- | --- | --- | --- | --- | --- | --- | --- | --- | --- | --- |
|  |  | mean | sem | n | mean | sem | n | mean | sem | n | normal distribution | test | Value | p |
| Fig. 6g | Amp. (mV) | 0.70 | 0.07 | 11 | 0.67 | 0.06 | 8 | 0.41 | 0.03 | 13 | yes | One-way ANOVA | F (2, 29) = 9.439, p=0.0007, followed by Tukey's multiple-comparisons test | p=0.0012 for PYN vs. PV; p=0.0083 for PYN vs. SST |
| Fig. 6g | Amp. (mV) | 0.70 | 0.04 | 3 | 0.65 | 0.09 | 3 | 0.41 | 0.02 | 4 |  | One-way ANOVA | F (2, 7) = 8.641, p=0.0129, followed by Tukey's multiple-comparisons test | p=0.0154 for PYN vs. PV; p=0.0414 for PYN vs. SST |
| Fig. 6h | I-E-I (s) | 0.15 | 0.02 | 11 | 0.65 | 0.21 | 8 | 0.56 | 0.12 | 13 | no | Kruskal‒Wallis test | H(2)=15.9, p=0.0004, followed by Dunn's multiple-comparisons test | p=0.0009 for PYN vs. PV; p=0.0039 for SST vs. PV |
| Fig. 6h | I-E-I (s) | 0.15 | 0.02 | 3 | 0.62 | 0.17 | 3 | 0.57 | 0.08 | 4 |  | One-way ANOVA | F (2, 7) = 6.122, p=0.0476, followed by Tukey's multiple-comparisons test | p=0.0364 for PYN vs. PV; p=0.0508 for PYN vs. SST |
| Fig. 6i | Freq. (Hz) | 7.15 | 0.93 | 11 | 3.08 | 1.23 | 8 | 2.44 | 0.34 | 13 | no | Kruskal‒Wallis test | H(2)=15.45, p=0.0004, followed by Dunn's multiple-comparisons test | p=0.0007 for PYN vs. PV; p=0.0033 for SST vs. PV |
| Fig. 6i | Freq. (Hz) | 7.23 | 0.75 | 3 | 3.02 | 0.58 | 3 | 2.44 | 0.22 | 4 |  | One-way ANOVA | F (2, 7) = 26.61, p=0.0005, followed by Tukey's multiple-comparisons test | p=0.0019 for PYN vs. PV; p=0.0006 for PYN vs. SST |
| Fig. 6k | count | 12.39 | 4.80 | 9 | 2.02 | 1.35 | 10 | 1.60 | 0.86 | 10 | no | Kruskal‒Wallis test | H(2)=9.57, p=0.0084, followed by Dunn's multiple-comparisons test | p=0.0429 for PYN vs. PV; p=0.006 for SST vs. PV |
| Fig. 6k | count | 12.39 | 2.96 | 3 | 1.95 | 0.99 | 3 | 1.67 | 0.67 | 3 | no | One-way ANOVA | F(2,6)=11.02, p=0.0098, followed by Tukey's multiple-comparisons test | p=0.0165 for PV vs. PYN; p=0.006 for PV vs. SST |

| Fig. 6 | measurement | veh. | | | MPX | | | statistics | | | |
| --- | --- | --- | --- | --- | --- | --- | --- | --- | --- | --- | --- |
|  |  | mean | sem | n | mean | sem | n | normal distribution | test | Value | p |
| Fig. 6m | count | 1.002 | 0.0048 | 7 | 0.781 | 0.071 | 7 | yes |  | t(6)=3.079 | 0.0217 |
| Fig. 6m | count | 1.002 | 0.0058 | 3 | 0.785 | 0.061 | 3 |  | Paired t test | t(2)=3.231 | 0.0839 |
| Fig. 6n | count at 50 µA | 0.952 | 0.7143 | 7 | 1.333 | 1.333 | 7 |  | Two-way ANOVA | F (1, 30) = 25.06, p< 0.0001 for treatment, followed by Sidak's multiple-comparisons test | >0.9999 |
|  | count at 100 µA | 14.33 | 2.9205 | 7 | 9.381 | 2.922 | 7 |  |  |  | 0.4919 |
|  | count at 150 µA | 26.38 | 6.5155 | 7 | 19.76 | 5.056 | 7 |  |  |  | 0.2025 |
|  | count at 200 µA | 39.24 | 11.625 | 7 | 29.24 | 9.099 | 7 |  |  |  | 0.0173 |
|  | count at 250 µA | 53.71 | 13.981 | 7 | 39.62 | 14.35 | 7 |  |  |  | 0.0005 |
| Fig. 6p | count | 0.999 | 0.0351 | 6 | 1.379 | 0.196 | 6 | no | Wilcoxon matched-pairs signed-rank test | W=21 | 0.0313 |
| Fig. 6p | count | 1.015 | 0.0428 | 3 | 1.345 | 0.049 | 3 |  | Paired t test | t(2)=3.658 | 0.0673 |
| Fig. 6q | count at 50 µA | 0.722 | 0.3268 | 6 | 0.722 | 0.505 | 6 |  | Two-way ANOVA | F (1, 30)=4.661, p=0.039 for treatment, followed by Sidak's multiple-comparisons test | >0.9999 |
|  | count at 100 µA | 4.278 | 1.5833 | 6 | 5.389 | 1.665 | 6 |  |  |  | 0.4628 |
|  | count at 150 µA | 6.722 | 1.9234 | 6 | 6.944 | 1.326 | 6 |  |  |  | 0.9997 |
|  | count at 200 µA | 8.111 | 1.546 | 6 | 8.111 | 1.104 | 6 |  |  |  | >0.9999 |
|  | count at 250 µA | 8.222 | 1.5508 | 6 | 9.444 | 0.687 | 6 |  |  |  | 0.3545 |
|  | count at 300 µA | 8.667 | 0.9813 | 6 | 9.556 | 0.542 | 6 |  |  |  | 0.7015 |

Data with a gray background represent statistical data based on values averaged across individual mice.

Supplemental Table 9, linked to Figure 7

| Fig. 7 | measurement | PV-2A-shRNA veh. | | | PV-2A-shRNA MPX | | | statistics | | | |
| --- | --- | --- | --- | --- | --- | --- | --- | --- | --- | --- | --- |
|  |  | mean | sem | n | mean | sem | n | normal distribution | test | Value | p |
| Fig. 7c | Duration in Center (s) | 50.90 | 7.71 | 8 | 39.54 | 11.69 | 8 | yes | unpaired t test | t(14)=0.8114 | 0.431 |
| Fig. 7d | Total distance (m) | 22.47 | 2.87 | 8 | 22.07 | 1.64 | 8 | no | Mann‒Whitney test | U=31 | 0.959 |
| Fig. 7e | Distance 0-5 min (m) | 12.58 | 1.33 | 8 | 11.83 | 0.84 | 8 | - | multiple *t* test | t(14)=0.4804 | 0.638 |
|  | Distance 5-10 min (m) | 9.89 | 1.58 | 8 | 10.24 | 1.24 | 8 | - |  | t(14)=0.1774 | 0.862 |
|  | Distance 10-15 min (m) | 9.32 | 1.08 | 8 | 10.44 | 1.52 | 8 | - |  | t(14)=0.5993 | 0.559 |
|  | Distance 15-20 min (m) | 8.23 | 1.48 | 8 | 8.70 | 2.04 | 8 | - |  | t(14)=0.1857 | 0.855 |
|  | Distance 20-25 min (m) | 6.73 | 1.18 | 8 | 9.70 | 1.96 | 8 | - |  | t(14)=1.296 | 0.216 |
|  | Distance 25-30 min (m) | 5.49 | 1.51 | 8 | 8.37 | 2.13 | 8 | - |  | t(14)=1.104 | 0.288 |
| Fig. 7f | Total distance (m) | 52.24 | 6.38 | 8 | 59.28 | 7.59 | 8 | yes | unpaired t test | t(14)=0.7098 | 0.490 |
| Fig. 7g | resp.65 dB | 18.75 | 3.81 | 8 | 15.14 | 1.58 | 8 | yes | unpaired t test | t(14)=0.8735 | 0.397 |
| Fig. 7h | resp.120 dB | 1040.00 | 94.04 | 8 | 918.00 | 132.50 | 8 | yes | unpaired t test | t(14)=0.7523 | 0.464 |
| Fig. 7i | 78 dB | 31.93 | 7.23 | 8 | 43.13 | 7.79 | 8 | - | Two-way ANOVA | F (1, 56) = 4.469, p=0.039 for treatment, followed by Two-stage linear step-up procedure of Benjamini, Krieger and Yekutieli | 0.269 |
|  | 82 dB | 47.00 | 5.77 | 8 | 59.45 | 7.90 | 8 | - |  |  | 0.220 |
|  | 86 dB | 51.04 | 6.15 | 8 | 57.99 | 7.40 | 8 | - |  |  | 0.491 |
|  | 90 dB | 51.95 | 7.38 | 8 | 63.79 | 6.86 | 8 | - |  |  | 0.243 |
| Fig. 7j | Immobile (s) | 120.00 | 12.51 | 8 | 141.50 | 14.41 | 8 | yes | unpaired t test | t(14)=1.131 | 0.277 |
